# Ex Situ Reconstruction-Shaped Ir/CoO/Perovskite Heterojunction for Boosted Water Oxidation Reaction

**DOI:** 10.1021/acscatal.2c05684

**Published:** 2023-03-29

**Authors:** Hongquan Guo, Yanling Yang, Guangming Yang, Xiaojuan Cao, Ning Yan, Zhishan Li, Emily Chen, Lina Tang, Meilan Peng, Lei Shi, Shunji Xie, Huabing Tao, Chao Xu, Yinlong Zhu, Xianzhu Fu, Yuanming Pan, Ning Chen, Jinru Lin, Xin Tu, Zongping Shao, Yifei Sun

**Affiliations:** † College of Energy, Xiamen University, Xiamen 361005, China; ‡ State Key Laboratory of Materials-Oriented Chemical Engineering, College of Chemical Engineering, Nanjing Tech University, Nanjing 211816, China; § School of Physics and Technology, Wuhan University, Wuhan 430072, China; ∥ Monash Centre for Electron Microscopy, Monash University, Victoria 3800, Australia; ⊥ Key Laboratory of Low-grade Energy Utilization Technologies and Systems, MOE, Chongqing University, Chongqing 40030, China; # School of Chemical Engineering, Dalian University of Technology, Dalian 116024, China; ¶ State Key Laboratory of Physical Chemistry of Solid Surface, Xiamen University, Xiamen 361005, China; ∇ College of Chemistry and Chemical Engineering, Xiamen University, Xiamen 361005, China; ○ Department of Electrical Engineering and Electronics, University of Liverpool, Liverpool L69 3GJ, U.K.; ⧫ Institute for Frontier Science, Nanjing University of Aeronautics and Astronautics, Nanjing 210001, China; †† Shenzhen Institutes of Advanced Technology, Chinese Academy of Sciences, Shenzhen 518055, China; ‡‡ Department of Geological Sciences, University of Saskatchewan, Saskatoon SK S7N 5E2, Canada; §§ Canadian Light Source, University of Saskatchewan, Saskatoon SK S7N 0X4, Canada; ∥∥ Key Laboratory of Pollution Ecology and Environmental Engineering, Institute of Applied Ecology, Chinese Academy of Sciences, Shenyang, Liaoning 110016, China; ● Shenzhen Research Institute of Xiamen University, Shenzhen 518057, China

**Keywords:** ex situ reconstruction, heterojunction, oxygen evolution, lattice oxygen mechanism, charge accumulation

## Abstract

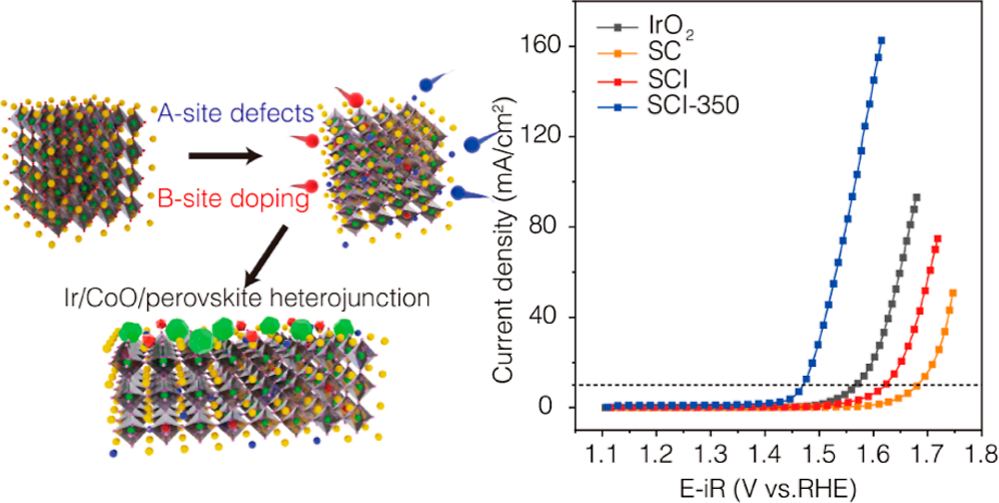

The oxygen evolution reaction (OER) is the performance-limiting step in the process of water splitting. In situ electrochemical conditioning could induce surface reconstruction of various OER electrocatalysts, forming reactive sites dynamically but at the expense of fast cation leaching. Therefore, achieving simultaneous improvement in catalytic activity and stability remains a significant challenge. Herein, we used a scalable cation deficiency-driven exsolution approach to ex situ reconstruct a homogeneous-doped cobaltate precursor into an Ir/CoO/perovskite heterojunction (SCI-350), which served as an active and stable OER electrode. The SCI-350 catalyst exhibited a low overpotential of 240 mV at 10 mA cm^–2^ in 1 M KOH and superior durability in practical electrolysis for over 150 h. The outstanding activity is preliminarily attributed to the exponentially enlarged electrochemical surface area for charge accumulation, increasing from 3.3 to 175.5 mF cm^–2^. Moreover, density functional theory calculations combined with advanced spectroscopy and ^18^O isotope-labeling experiments evidenced the tripled oxygen exchange kinetics, strengthened metal–oxygen hybridization, and engaged lattice oxygen oxidation for O–O coupling on SCI-350. This work presents a promising and feasible strategy for constructing highly active oxide OER electrocatalysts without sacrificing durability.

## Introduction

The increasing consumption of fossil fuels has led to a rise in CO_2_ emissions, necessitating the need for the development of renewable energy sources (RESs).^1^ However, the intermittent and seasonal nature of RES remains a significant challenge to their integration into the electricity grid. One promising method is to convert and store these energies as H_2_ through electrolysis.^2^ Nowadays, water electrolyzers are the most attractive technology for producing H_2_ in the cathode [hydrogen evolution reaction (HER)] and O_2_ in the anode [oxygen evolution reaction (OER)] simultaneously via electrolysis. However, compared to HER, which involves a two-electron-transfer process, the OER is a relatively sluggish reaction that involves multi-step electron–proton coupling transfer and energy-intensive O–O bonding, making it more thermodynamically and kinetically unfavorable.^3,4^ Therefore, developing OER electrocatalysts that promote charge transfer and facilitate the reformation of O-containing chemical bonds is crucial.

In electrocatalysis, efficient electrocatalytic conversion requires the adsorption and storage of charged reactants.^5^ The accumulation of charge on the electrocatalyst has been identified as a cause of the evolution of surface reaction sites.^6^ Recent work has also illustrated that the charging of catalyst surfaces influences the formation and rupture of chemical bonds, and the activation free energy of OER is closely related to the amount of surface charge on electrocatalysts,^7^ confirming the importance of charge accumulation in OER. On the other hand, it is also important to construct reactive sites with high intrinsic catalytic activity. So far, many precious/transition metal oxide (such as IrO_2_,^8^ NiO,^9,10^ and CoO^11,12^) electrocatalysts were reported to be effective for OER.^13^ Alternatively, the development of perovskite oxides by diluting and redispersing reactive metal sites (typically B-sites) in an ordered oxide framework is a promising approach for improving performance.^14,15^ The monoclinic SrIrO_3_ in alkaline electrolytes demonstrated a low overpotential of 300 mV to achieve 10 mA cm^–2^, significantly lower than that of IrO_2_.^16^ The hydrated potassium-inserted LiIrO_3_-layered compound upon reaction with KOH was also reported as an effective OER electrocatalyst with a stabilized reaction surface.^17^ Recently, plenty of studies have highlighted that the surface lattice oxygen in perovskite oxide can also participate in the fast alkaline OER process via a lattice oxygen mechanism (LOM) rather than a traditional adsorbate evolution mechanism (AEM).^18,19^ Grimaud et al., discovered the importance of electrophilic active surface oxygen species (O^–^) in La_2_LiIrO_6_ (LLI) during OER. The cation leaching-induced valance change and participation of activated lattice oxygen atoms acted as a trigger to boost the OER activity.^20^ However, the activation of lattice oxygen usually cannot avoid the entanglement of lowered transition-metal oxide coordination, resulting in a dynamic cation dissolution.^21−23^ Hence, a tradeoff is usually observed in numerous material systems, where the catalyst reactivity enhancement due to lattice oxygen is counterbalanced by its long-term stability and structural integrity, especially in the perovskite oxide system for a scalable water electrolysis apparatus.^24,25^ This conclusion was supported by the observation of fast deactivation of LLI.^20^

To address the aforementioned formidable challenges, in this work, we conceived an OER electrocatalyst consisting of an ex situ-formed Ir/CoO/perovskite heterojunction derived from a doped cobaltate precursor. The fully exposed Ir nanoclusters and CoO nanoparticles anchored perovskite was assembled following the design principles of exsolution and A-site deficiency,^26^ which not only demonstrates an exponentially larger electrochemical surface area for charge storage, but also provides a reactive/durable platform for OER. The as-prepared heterojunction requires only a low overpotential of 240 mV at 10 mA cm^–2^ in 1 M KOH and has long durability of over 150 h in the membrane electrode assembly (MEA) mode. By combining theoretical approaches and trustworthy spectroscopy results, we confirmed that the expedited lattice oxygen redox and facilitated direct O–O coupling on such catalytically active heterojunction via the LOM pathway can be attributed to the accelerated oxygen exchange rate and optimized band structure with strong metal–oxygen hybridization. This work provides new insights into the development of practical and efficient catalysts for water oxidation or other heterogeneous catalysis involving metals and lattice oxygen.

## Results and Discussion

### Structure Characterizations

**Figure 1:**
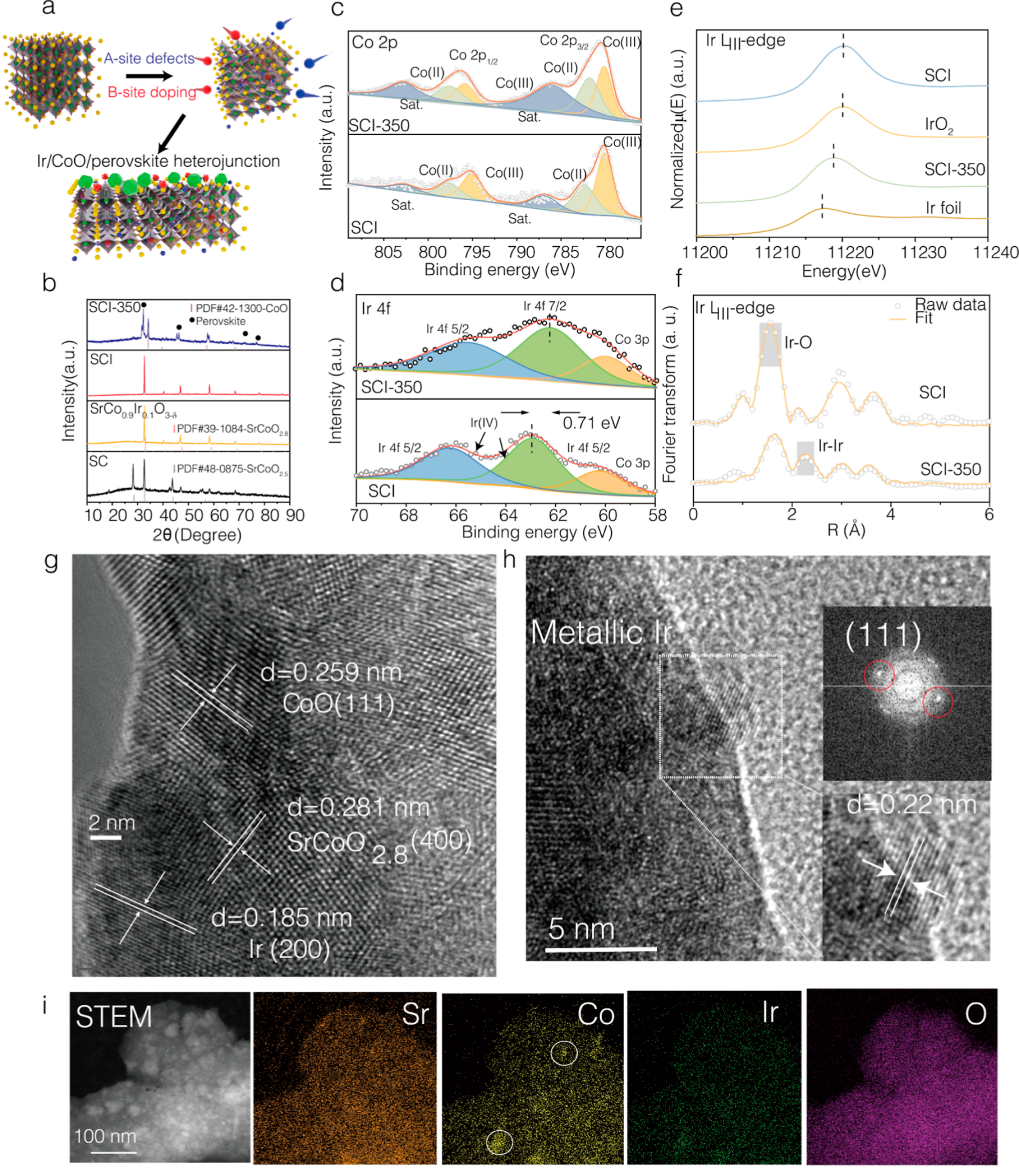
(a) Fabrication schematic of SCI-350 electrocatalyst. Red presents Ir; green presents Co; yellow presents Sr; blue presents A-site; pink (small) presents O; green pyritohedron presents CoO; and red deltahedron presents Ir clusters. (b) XRD patterns of various electrocatalysts. (c,d) Co 2p and Ir 4f XPS spectra of SCI and SCI-350. (e) XANES spectra of Ir foil, IrO_2_, SCI, and SCI-350 measured at the Ir L_III_-edge. (f) *k*^3^-weighted Ir L_III_-edge EXAFS spectra of SCI and SCI-350. (g,h) HR-TEM images of SCI-350 (the PDF card number for metallic Ir is PDF#06-0598). (i) high-angle annular dark field scanning transmission electron microscopy (HAADF–STEM) and energy-dispersive X-ray (EDX) mapping images of SCI-350.

Figure 1a depicts the schematic of the composition evolution of the electrocatalysts during our fabrication procedure. We characterized their phase information synchronously by analyzing X-ray diffraction (XRD) patterns (Figure 1b). The hexagonal SrCoO_3−δ_ (SC) and tetragonal Ir-doped SrCoO_3−δ_ (SrCo_0.9_Ir_0.1_O_3−δ_) perovskites were initially prepared and characterized.^27,28^ Additionally, a 10 mol % A-site deficiency was introduced to form tetragonal Sr_0.9_Co_0.9_Ir_0.1_O_3−δ_ (SCI) with a space group of *P*4/*mmm* (the refined results are shown in Figure S1). The role of Ir incorporation in the crystal phase becomes prominent when exposed to a reducing atmosphere (5% H_2_/N_2_, 350 °C for 3 h). A previous work has illustrated that the A-site deficiency in the perovskite facilitated the phase segregation of B-site cations from a thermodynamic aspect.^28,29^ However, after being reduced under the same conditions, the peaks in the diffraction patterns of Sr_0.9_CoO_3−δ_ with A-site deficiency only become weaker and broader, without the formation of noticeable impurities, which can be ascribed to a loss of long-term ordering and a decrease in crystallinity (Figure S2). In comparison, even when the octahedron-coordinated Co sites were substituted by Ir, the material without an A-site deficiency demonstrates significant phase separation following reduction, as confirmed by the diffraction peak of CoO (Figure S3). In contrast, the material with both Ir doping and A-site deficiency (SCI-350) presents the co-existence of CoO and distortion of the perovskite lattice (Figure 1b). However, no metallic iridium is observed in XRD patterns, which could be due to the small particle size being below the detection limitation of the instrument. The (110) diffraction peak shifted from 32.66 to 32.34°, suggesting the expansion of the unit cell. Meanwhile, the splitting of the XRD diffraction peaks can be observed, which could be explained by a decrease in the tetragonal symmetry and the formation of oxygen vacancy ordering.^30,31^

In addition, we investigated the chemical state evolution of the electrocatalyst during preparation using X-ray photoelectron spectroscopy (XPS) (Figures 1c,d and S4). The presence of Sr, Co, Ir, and O elements in both SCI and SCI-350 could be confirmed by the XPS survey scan, as shown in Figure S4a,b. Figure 1c,d shows the Ir 4f and Co 2p spectra of SCI and SCI-350, respectively. The binding energy peaks at 62.9 (4f_7/2_) and 66.2 eV (4f_5/2_) can be assigned to Ir (IV), which stem from Ir cations in the SCI perovskite phase.^28^ The binding energy peak of Co 3p also emerges concurrently. After reduction, the position of the binding energy peak of Ir shifted downward by ∼0.71 eV for SCI-350, suggesting a decrease in the average oxidation state of Ir. It is most likely due to the formation of metallic Ir.^32^ According to the Co 2p of SCI, the Co^3+^ 2p_3/2_, Co^2+^ 2p_3/2_, Co^3+^ 2p_1/2_, and Co^2+^ 2p_1/2_ peaks appear at 780.1, 782.3, 795.1, and 797.6 eV, respectively. The 2p_3/2_/2p_1/2_ satellite peaks can be found at 786.8 and 802.6 eV, respectively. Meanwhile, the average valence state of Co in SCI was calculated to be ∼2.61, while it was ∼2.50 in SCI-350 (Table S3-3). These results indicate the reduced oxidation state of Co after reduction, which is consistent with the XRD data.^33^ The overall oxidation state of Ir in various electrocatalysts was further characterized by X-ray absorption spectroscopy (XAS), as shown in Figure 1e. The spectra of SCI with an Ir oxidative state of ∼4+ and the metallic Ir reference are presented as well.^28^ The spectra of SCI-350 are located in the middle, implying that the majority of Ir cations were deeply reduced. The experimental and fitted Fourier transformed (FT) Ir L_3_-edge extended X-ray absorption fine-structure (EXAFS) spectra of SCI and SCI-350 are shown in Figure 1f (raw data in dot lines and fitting curve in red lines). Detailed structural parameters obtained from the least-squares fits of the EXAFS data are summarized in Table S1. Specifically, the first peak in SCI is ascribed to the contribution from Ir–O backscattering with the coordination number (CN) of 5.8(5) at an averaged interatomic distance of 1.94(1), suggesting an IrO_6_ octahedral structure.^28^ After reduction, a reduced peak intensity is observed in SCI-350. The fitting results show that SCI-350 has a lower CN [3.6(1)] and a larger Debye–Waller factor for the Ir–O path compared to SCI, suggesting the under-coordinated nature and distortion of IrO_6_ octahedra after reduction. In addition, another peak structure shown in the R-space data of SCI-350 is ascribed to the Ir–Ir backscattering (located between the Ir–O and Ir–Sr shells) with a fitted interatomic distance of 2.65(3) Å, which falls into the range matching the bond length of the Ir–Ir shell in metallic Ir nanoparticles.^34^ However, this position did not perfectly match that of the Ir–Ir bond length in standard Ir foil [Figure S5, interatomic distance of 2.70(1) Å], which may be due to its small particle size and strong electronic interaction with the perovskite support in the exsolved system. These results agree with the transmission electron microscopy (TEM) images (vide infra), confirming the emergence of Ir nanoclusters on the perovskite.

The high-resolution TEM (HR-TEM) image of SCI (Figure S6a) displays clear lattice fringes with a *d*-spacing of 0.271 nm, corresponding to the (400) plane of perovskites. Moreover, the energy-dispersive X-ray (EDX) mapping results under high-angle annular dark field scanning transmission electron microscopy (HAADF–STEM) revealed the uniform distribution of Sr, Co, Ir, and O elements in SCI (Figure S6b). Table S3-1 and S3-2 shows the experimentally measured and calculated weight content of Ir in various electrocatalysts. The Ir content in SCI-350 is 75% lower by weight compared to that in the benchmark catalyst IrO_2_. Figure S6c,d suggests the observable agglomeration of SCI during the thermal reduction treatment at 350 °C. However, the co-existence of CoO, Ir, and perovskite can be clearly confirmed in the HR-TEM images (Figure 1g,h). The lattice fringes with *d*-spacings of 0.259 and 0.22 nm can be identified, corresponding to the (111) plane of CoO (the particle size is ∼10 nm) and the (111) plane of metallic Ir (the particle size is ∼3 nm), respectively. The EDX mapping results under the HAADF–STEM (Figure 1i) mode show the segregation of CoO nanoparticles and highly dispersed Ir pinned on the perovskite support.

### OER Performance

The OER performances of various electrocatalysts were first evaluated using linear sweep voltammetry (LSV) in a three-electrode configuration, with the Hg/HgO reference electrode calibrated before measurement (Figure S7). To optimize the phase-structure composition, the contents of Ir and Sr were systematically screened, considering the tradeoff between structure integrity and performance (Figures S8 and S9). As a result, we can conclude that Sr_0.9_Co_0.9_Ir_0.1_O_3−δ_ outperformed other counterparts. Furthermore, the structure-optimized Sr_0.9_Co_0.9_Ir_0.1_O_3−δ_ was treated under different reducing conditions to induce the formation of the Ir/CoO moiety on the surface of perovskites (Figure S10).

**Figure 2:**
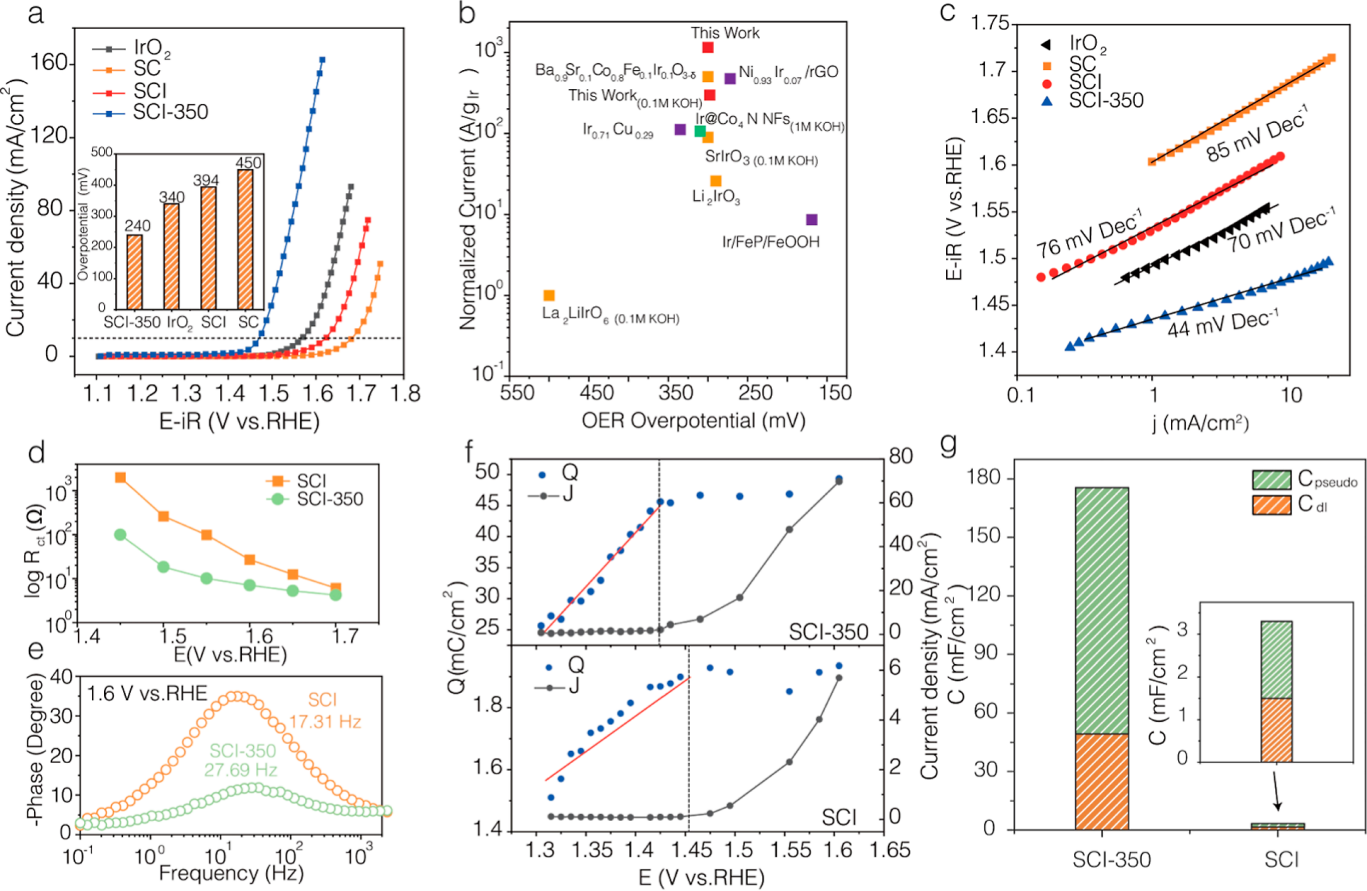
(a) LSV curves of various electrocatalysts in 1 M KOH electrolyte. (b) Comparison of Ir MA of representative Ir-based oxide electrocatalysts. The electrocatalysts were measured in 1 M KOH with specific notation shown in the Figure. (c) Tafel slope plots. (d) Response of the total charge transfer resistance (*R*_total_) vs applied potential. (e) EIS–Bode plots at 1.6 V vs RHE. (f) *Q* vs *E* and *J* vs *E* plots calculated from the pulse voltammetry. (g) Comparison of the pseudocapacitance and double layer capacitance.

According to the H_2_-temperature-programmed reduction (TPR) results (Figure S11), a temperature higher than 300 °C is required to initiate the co-reduction of Co and Ir cations. The Brunauer–Emmett–Teller (BET) surface area results (Table S4) indicate that all samples show a similar surface area of less than 1 m^2^ g^–1^, which minimizes their contribution to the apparent OER performance. SCI-350 has a low OER overpotential of 240 mV at a geometric current density of 10 mA cm^–2^ (η_10_), which is significantly lower than that of benchmark IrO_2_ (340 mV), SCI (394 mV), and SC (450 mV), demonstrating the superior OER activity of SCI-350 (Figure 2a). The OER activity of SCI-350 is superior to other catalysts in this study and most of the recently reported Ir-based electrocatalysts, in terms of both apparent activity and normalized Ir mass activity (MA) (Figure 2b, Table S2-1 and S2-2). Furthermore, the catalyst MA of different catalysts at a potential of 1.47 V (Figure S12) also indicates that SCI-350 has a superb MA of 19.5 A g_catalyst_^–1^, which is orders of magnitude higher than SCI (MA = 0.41 A g_catalyst_^–1^) and SC (MA = 0.07 A g_catalyst_^–1^).

To gain more insights into the reaction kinetics, the Tafel plots are displayed in Figure 2c. The Tafel slopes of SCI-350, SCI and SC are ∼44, 76 and 85 mV dec^–1^, respectively, implying that the intrinsic reaction kinetic rate is in the order of SCI-350 > SCI > SC. Moreover, the electrochemically active surface area (ECSA) was calculated using double-layer capacitance (*C*_dl_) plots (Figures S13 and S14). The *C*_dl_ is 49.2 mF cm^–2^ for SCI-350, indicating its desirable capability for charge accumulation. In comparison, SCI only has a negligible *C*_dl_ of 1.5 mF cm^–2^, emphasizing the prominent charge storage capability of the heterojunction. Based on these results, the calculated ESCA for SCI-350 is 1230 cm^2^, more than 30 times that of SCI (37.5 cm^2^). Figure S15 depicts the ECSA-normalized specific activity (SA) at *E* = 1.5 V. The SCI-350 (SA = 29 μA cm^–2^) still offers a much higher SA than the SCI (SA = 15.2 μA cm^–2^).

Electrochemical impedance spectroscopy (EIS) is a powerful in situ technology to probe the reaction kinetics as well as the electrochemical properties of the interfaces. Operando EIS measurements were performed in 1 M KOH at different potentials ranging from 1.30 to 1.70 V (Figure S16) to gain a thorough understanding of the OER kinetics. The obtained Nyquist plots were fitted with equivalent circuits shown in the inset, and the values of each circuit component are shown in Tables S5 and S6. *R*_s_ represents the resistance from the electrolyte and all samples had similar *R*_s_ values of ∼6 Ω. Because *R*_1_ has a nearly constant value of ∼1 Ω, the high-frequency semicircle (*R*_1_||CPE_1_) is potential independent. Therefore, it is not a direct measure of OER kinetics but rather an indication of the electrode porosity.^35,36^ The *R*_ct_ represents the charge-transfer resistance for the whole OER process, and CPE_2_ is ascribed to the double layer capacitance (*C*_dl_) of the electrodes, respectively.^37−39^ Catalysts with smaller *R*_ct_ render faster charge-transfer kinetics. As shown in the summarization plot in Figure 2d, Tables S5 and S6, *R*_ct_ of SCI-350 is much lower than that of pure SCI (1.4–1.7 V), demonstrating much faster kinetics in the adsorption of OH ions during OER. For example, at a potential of 1.55 V, the *R*_ct_ of SCI-350 is 10.1 Ω, which is more than 9 times lower than that of SCI. Furthermore, as a complementary evidence, the peak frequency of the EIS–Bode plot (Figure 2e) was measured to reflect the time scale for interfacial charge transfer.^33^ At 1.6 V, the Bode plots of SCI-350 deliver a higher frequency of 27.69 Hz (compared to a SCI of 17.31 Hz) with a lower phase angle, which is consistent with the Tafel slope results.

To accurately quantify the overall accumulated charge (both double-layer capacitance and pseudocapacitance) on the surface of electrocatalysts, the pulse voltammetry measurement was performed (Figure S17). The electrocatalyst was initially set to a specific positive potential (1.305 to 1.605 V) and before being abruptly switched to the open circuit potential (OCP). The corresponding cathodic transient current peak was monitored and integrated to determine the accumulated charge. Figure 2f summarizes the relationship between the accumulated positive charge (*Q*), OER current density (*J*), and the applied potential (*E*), and all electrocatalysts exhibit a similar shape of *Q*–*E* and *J*–*E* curves. All of the curves exhibit two distinct potential regions separated by the onset potential of OER. At lower potentials, where the OER is not initialized, the current is predominantly contributed by surface charges, and the electrocatalysts exhibit a linear relationship between *Q* and *E*. After reaching the OER onset potential, the OER current starts to increase, and the accumulated charge becomes nearly constant as the applied potential increases. The slope of (∂*Q*/∂*E*) in the linear region can be used to calculate the charge storage capacity of the electrocatalysts, yielding a capacitance value of 175.5 mF cm^–2^ for SCI-350 and 3.3 mF cm^–2^ for SCI (Figure 2g). Therefore, the pseudocapacitances of SCI-350 and SCI are 126.3 and 1.8 mF cm^–2^, respectively. The substantial charge accumulation is responsible for the increased surface coverage of reactants, which further facilitates the OER kinetics.

### Origin of High Performance of Heterojunction

**Figure 3:**
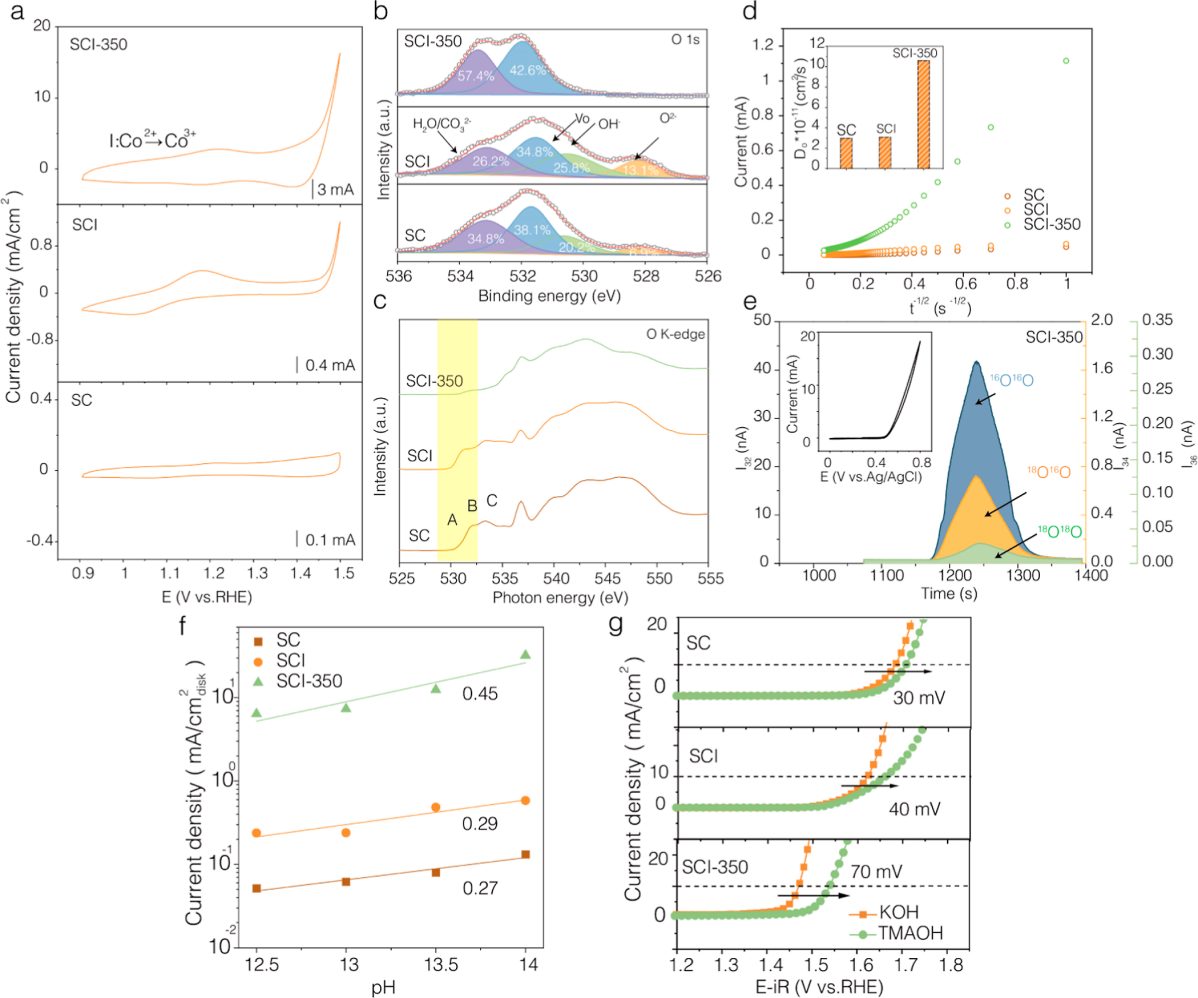
(a) CV curves within the Co redox potential range at a scan rate of 5 mV/s in 1 M KOH. (b) O 1s XPS spectra. (c) O K-edge XANES plots. (d) Oxygen diffusion coefficient experiments of various electrocatalysts. (e) DEMS signals of ^16^O_2_ (I_32_), ^16^O^18^O (I_34_), and ^18^O_2_ (I_36_). The inset shows the cyclic voltammograms during the DEMS measurement. (f) Current densities of various electrocatalysts at 1.5 V vs RHE as a function of the pH value. (g) Polarization curves of different electrocatalysts in 1 M KOH and 1 M TMAOH electrolytes, respectively.

These findings show that the heterojunction of Ir/CoO/perovskites has enhanced charge accumulation in terms of both *C*_pseudo_ and *C*_dl_. Even when treated in the same way, the reduced Sr_0.9_CoO_3−δ_ perovskite showed a lower *C*_dl_ of 20.2 mF cm^–2^ and poorer activity with an overpotential of 390 mV (Figure S18). This result demonstrates that ex situ formed Ir/CoO dispersed on a perovskite matrix plays a predominant role in increasing electrochemical active surface area and intrinsic activity. To better understand the formation and evolution of the dynamic reactive sites on electrocatalysts, we further employed the detailed cyclic voltammetry (CV) measurements to investigate the dynamic structural evolution of various electrocatalysts. Metallic Ir^40^ or IrO_2_ (Figure S19) has a small pseudocapacitive feature due to the redox behavior of Ir(III/IV/V) with either anodic or cathodic current intensity lower than 0.01 mA cm^–2^. In our cases, the potential region is below an equilibrium potential of OER of 1.23 V versus reversible hydrogen electrode (RHE) and the onset of OER. According to previous works, the CV results of SCI-350 show anodic I peaks (Figure 3a), which could be attributed to the oxidation of Co(II) (CoO in our case) to Co(III) (i.e., CoOOH see in situ Raman data below), and the peak associated with the oxidation of Co(III) to Co(IV) is invisible.^41,42^ Frei and co-workers reported that Co(III) species act as an initiator of the OER process.^43^ Meanwhile, the current intensity of this redox peak is more than 1 order of magnitude higher than that of SCI, which is consistent with the results of the charge storage analysis in Figure 2g.^44^

Because the oxygen state in electrocatalysts is strongly related to the O–O coupling process, the chemical valence of O on the surface was investigated using XPS. The O 1s spectra of different materials (SC, SCI, and SCI-350) in Figure 3b could be deconvoluted into four peaks. The binding energy at ∼528.3 eV (P1) can be ascribed to lattice oxygen species (O^2–^). Peaks at ∼530.4 (P2), ∼531.5 (P3), and ∼533 eV (P4) can be attributed to adsorbed oxygen/hydroxyl species (O_2_/OH^–^), oxygen vacancy-related species and adsorbed water (H_2_O) or carbonates (CO_3_^2–^), respectively.^45^ Meanwhile, it is generally recognized that the H_2_O/C groups are linked to hydrophilic properties.^46,47^ The reduction process eliminates the peak corresponding to lattice oxygen (P1) and increases the concentration of oxygen vacancies (P3). Meanwhile, the area ratio of P4 increased from 26.2 to 57.4%, suggesting the enhanced hydrophilicity of the materials, which is beneficial for achieving faster kinetics of OER. As XPS is a relatively surface-sensitive tool with a penetration depth of less than 10 nm, representative O K-edges XAS spectra were performed on all three electrocatalysts, complementing the collection of overall electronic configuration information. The main change in the O K-edge spectra upon reduction treatment takes place in the pre-edge, as highlighted by the shaded region in Figure 3c, where three distinct features labeled “A”, “B”, and “C” can be found. The feature “A” corresponds to the transition from O 1s to the ligand hole (L) in the 3d orbital, representing the number of ligand holes in O 2p orbitals. Moreover, the other two features (feature “B” and “C”) can be assigned to the unoccupied t_2g_ and e_g_ states from 3d orbitals, respectively.^48^ Compared to SCI and SC, the pre-edge peak of SCI-350 tapers off significantly, indicating a more reduced chemical state and the presence of more oxygen vacancies.

CV experiments were conducted in the 6 M KOH electrolyte and the surged quantity of oxygen vacancies (Figure S20a), and the oxygen-ion diffusion coefficients (*D*_O_) of SC, SCI, and SCI-350 were determined using chronoamperometry (Figures 3d, S20b–d and Table S7). The *D*_O_ value of SCI at room temperature was calculated to be 3.08 × 10^–11^ cm^2^ s^–1^, which is comparable to SC. Remarkably, SCI-350 had a much higher diffusion coefficient of *D*_O_ = 10.58 × 10^–11^ cm^2^ s^–1^, which is more than three times larger than that of SCI (Table S7). The accelerated oxygen-ion diffusion in SCI-350 is believed to be related to the increased oxygen vacancy concentration.^49,50^ Therefore, it is typically expected to facilitate lattice oxygen participation during OER operation via the LOM pathway.^49,50^ Moreover, the presence of a large number of vacant oxygen sites could improve electrical conductivity, which benefits the charge transfer of OER. To validate our hypothesis, we performed an ^18^O isotope-labeling pretreatment, followed by in situ differential electrochemical mass spectrometry (DEMS) measurements on the ^18^O-labeled SCI-350. The results show remarkable signals of *m*/*z* = 32, *m*/*z* = 34, and *m*/*z* = 36 (Figure 3e), suggesting the presence of ^16^O_2_, ^16^O^18^O, and ^18^O_2_ during oxygen evolution. This result implies that SCI-350 follows the LOM mechanism,^51^ which collectively activates direct lattice oxygen coupling or the joint participation of lattice oxygen and oxygen from the OH group.

Typically, the electrocatalyst following LOM exhibits pH-dependent activity.^52^ Raising the pH can change the energy of the adsorbed intermediates, or it can increase the surface coverage or the OH concentration, resulting in the increased OER activity. Our experimental studies confirm an increase in OER activity for all samples when increasing pH from 12.5 to 14 (Figure S21), indicating that OER kinetics and thus LOM participation are pH dependent. Figure 3f further compares the OER activity of all electrocatalysts at 1.50 V vs RHE as a function of pH, with the slopes  being 0.45, 0.29, and 0.27 for SCI-350, SCI, and SC, respectively. Therefore, the as-formed Ir/CoO/perovskite heterojunction has a higher tendency for its lattice oxygen to participate in the OER. On the other hand, the OER via LOM involves the formation of negative oxygenated species [peroxo-like () and superoxo-like (O^2–^)] that can be captured by tetramethylammonium cations (TMA^+^). Accordingly, we compared the OER activities of different electrocatalysts in 1 M KOH and TMAOH solutions. As shown in Figures 3g and S22, the SCI-350 significantly increases OER overpotential by 70 mV at 10 mA cm^–2^, and its Tafel slope also increases by 24 mV dec^–1^, mainly due to the inhibition of the LOM.^53,54^

To shed more light on the detailed OER mechanism, density functional theory (DFT) calculations were performed to elucidate the electronic structures of the electrocatalysts. The structure models (Figures S23 and S24) for DFT calculations were developed based on the material characterization. Both the AEM and the LOM were considered as possible OER pathways. Additionally, because the potential at the OER region is highly oxidative, we constructed the IrO_2_/CoOOH/SrCoO_3_ model to represent the possible surface reconstruction.

The structure models of SCI-350 are shown in Figures S25 and S26. The step-by-step free energy diagrams were then calculated. For AEM, the reaction started from the adsorption of OH*, followed by sequential deprotonation to form O*, O–O bonding to generate OOH*, and desorption to produce oxygen. For LOM, the reaction proceeds via M–OH, (Vo)M–OO, Vo–M–OH, and OH–M–OH, in that order. The rate-determining step (RDS) at *U* = 0 V of SCI [Ir site on (110) facet] was the step from O* to OOH* (AEM) with a free energy of 0.58 eV, and the step from (Vo)M–OO to Vo–M–OH with a free energy of 0.45 eV (LOM). In comparison, the *OOH → *OO and O_V_–*OH → OH–*OH are the rate-determining steps for the AEM and LOM, respectively. Moreover, the SCI-350 favors the OER via LOM due to its lower free energy of 0.33 eV for RDS (Figure S27a,b). The DFT-calculated OER energy barrier agrees well with the Tafel slopes, EIS–Bode plots, as well as previous data, clarifying the high intrinsic OER kinetics of the Ir/CoO/perovskite hybrid-structure.

Meanwhile, projected density of state (PDOS) analysis was performed on the proposed atomic structures to elucidate the electronic structure (Figures S27c and S28). Previous studies illustrated that the energy difference between the metal 3d and O 2p-band centers determines the metal–oxygen covalency.^55^ By integrating the PDOS, our calculation results (Figure S27d) show that the distance between the metal 3d and O 2p centers for SCI-350 is 0.835 eV, which is much closer than for SCI (1.226 eV). The enlarged metal–oxygen covalency can promote electron transfer between metal and oxygen adsorbates, accelerating the OER rate.^56^ Moreover, as metal–oxygen covalency increases, the OER mechanism shifts from a concerted proton–electron-transfer pathway (AEM) to a decoupled proton–electron-transfer pathway (LOM), which agrees with our experimental and theoretical results. On the other hand, the O 2p band center on SCI-350 (Figure S27e) approaches the Fermi level (−0.571 eV), indicating a much lower oxygen-vacancy formation energy and accelerated oxygen exchange kinetics, facilitating intermediate uptake and ion transportation.^57^

**Figure 4:**
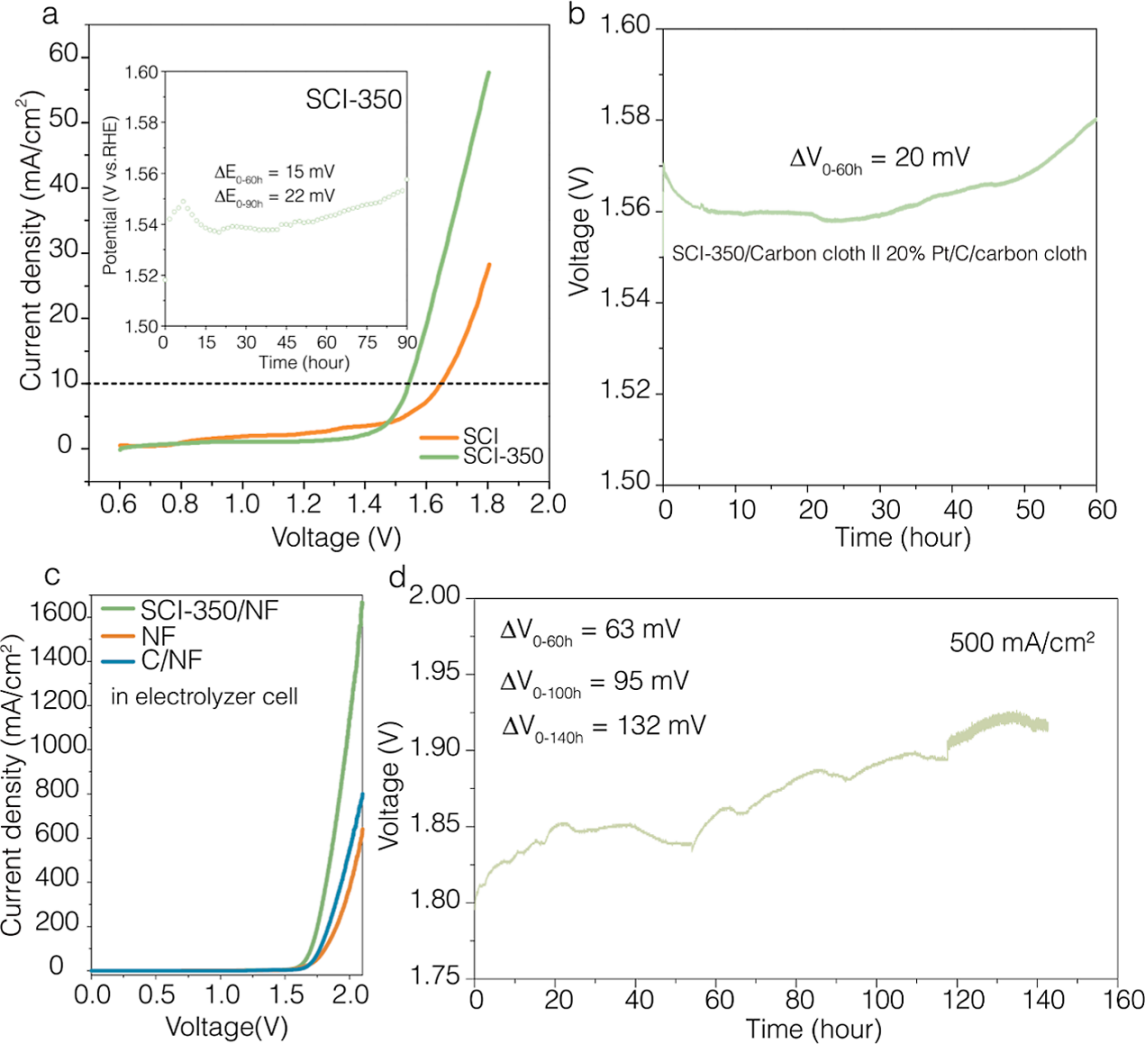
(a) LSV curves of the electrolyzer equipped with an OER electrocatalyst in a 1 M KOH solution. The inset shows a durability test in a three-electrode configuration under a 1 M KOH solution. (b) Stability test in the two-electrode configuration under 1 M KOH solution. (c) LSV curve of the SCI-350/NF, bare NF and C/NF in the water electrolysis cell (the voltage was iR-correct) under 6 M KOH solution. (d) Durability test in water electrolysis single cells. Cell voltage (the voltage was iR-corrected) at 500 mA cm^–2^. The voltage change value (Δ*V*_0–60h_) is the difference between the highest voltage and the lowest voltage in 60 h.

Finally, the durability test of SCI-350 was conducted in the galvanostatic mode at 10 mA cm^–2^ for 90 h, and the OER potential showed a decay of 22 mV (inset of Figure 4a). The two-electrode setup was then used to investigate the stability of both the SCI and SCI-350 catalysts (Figure 4a). The LSV curve in 1 M KOH shows that the (−) Pt/C/carbon cloth||SCI-350/carbon cloth (+) electrolyzer can deliver a current density of 10 mA cm^–2^ at a voltage of 1.52 V, which is significantly superior to the electrolyzers using SCI (1.63 V) as the OER catalyst. The electrolyzer voltage was measured at a current density of 10 mA cm^–2^ for at least 60 h of continuous testing (Figure 4b), indicating a variation of 20 mV. This fluctuation could be attributed to carbon corrosion. To further demonstrate the scalability and feasibility of our OER electrocatalyst for industrial applications, a zero-gap alkaline water electrolyzer was assembled using cobalt phosphide as the cathode (Figure S29). The configuration of the electrolyzer is (+) SCI-350||6 M KOH (25 °C), polyethersulfone separator||cobalt phosphide (−). The SCI-350 exhibited a cell voltage of 1.69 V at a lower current density of 100 mA cm^–2^ and a voltage of 1.95 V at a higher current density of 1000 mA cm^–2^ (25 °C with iR compensation), as presented in Figure 4c and Table S8. Figure 4d shows the cell voltage of the assembled water electrolysis cell with the SCI-350 electrode at an industrial-relevant current density of 500 mA cm^–2^, and the cell voltage showed an increase of 95 mV after 100 h.

To understand the cation leaching effect of SCI-350, we used ICP to quantify the concentration of dissolved metallic cations in the electrolyte during electrolysis. We conducted an independent durability test of SCI-350 in the galvanostatic mode at 20 mA cm^–2^ for 10 h (Figure S30a) and performed ICP measurements at different electrolysis times. As shown in Figure S30b,c, the leaching rates of Sr, Co, and Ir are 105.14, 0.94, and 6.21 ppb h^–1^, respectively, indicating that the loss of catalytically active element Ir and Co is much slower than that of Sr. The XRD pattern and scanning electron microscopy (SEM) images (Figure S31a–g) show that the post-OER SCI-350 (the one after the stability test mentioned above) still preserves the original perovskite crystal structure and surface morphology. The broad diffraction peak at ∼25° could be ascribed to carbon. The peak of CoO has disappeared, and the peak of CoOOH was not observed in the XRD pattern. However, the existence of CoOOH could be evidenced by the results of in situ Raman measurement (Figure S32), which indicates a transition from Co (II) to Co (III). Therefore, the undetectable XRD diffraction peak of CoOOH could be due to its amorphous phase. The deconvoluted high-resolution Co 2p, Ir 4f, Sr 3d, and O 1s XPS spectra and calculated average oxidative state of post-OER SCI-350 samples are shown in Figure S33 and Table S9. As expected, the oxidation of Co and Ir is observed after the prolonged OER measurement. The average oxidation state of Co is ∼2.67, which is higher than that of the fresh SCI-350 sample (∼2.50). Compared with pristine SCI-350, the position of the binding energy peak of Ir upshifts to high binding energy by ∼0.38 eV for post-OER SCI-350, suggesting an increase in the average oxidation state of Ir.

## Conclusions

This work reports on an ex situ reconstruction approach for preparing Ir/CoO/perovskite heterojunctions as active and durable OER catalysts. Both A-site deficiency and exsolution design principles were considered to achieve the optimal composition of the catalyst. The as-synthesized SCI-350 exhibited a low overpotential of 240 mV at 10 mA cm^–2^ and demonstrated promising potential for use in practical water splitting electrolyzes, maintaining a stable current density of 500 mA cm^–2^ for over 150 h. Detailed electrochemical analysis confirmed an order of magnitude increase in charge accumulation capability (from 3.3 to 175.5 mF cm^–2^). Furthermore, the combined XAS, XPS, and in situ Raman data demonstrated the presence of a large amount of vacant oxygen, which facilitated lattice oxygen diffusion and accelerated oxygen exchange during OER. The ^18^O isotope-labeling DEMS and DFT calculations further demonstrated the lattice oxygen activity for O–O coupling due to the strengthened metal–oxygen covalency and upshift of the O 2p center relative to the Fermi level. Our finding will shed light on the rational design of highly efficient oxide-based OER catalysts via the LOM pathway.

## Experimental Section

### Chemicals and Reagents

Strontium carbonate (SrCO_3_, 99%), cobalt (II,III) oxide (Co_3_O_4_, 99.9%), iridium oxide (IrO_2_, 99.99%), and 1 M KOH solution with H_2_^18^O (98 atom % H_2_^18^O) was purchased from Shanghai Energy chemical. Potassium hydroxide (KOH, ≥95%) and tetramethylammonium hydroxide pentahydrate (TMAOH, ≥97%) were purchased from Macklin. 20 wt % Pt/C, carbon paper, and Ni foam (purity 99.8%, thickness 1 mm and porosity 95%) were purchased from Sinero. 5 wt % Nafion solution was purchased from DUPONT. Isopropanol (≥99.7%) was purchased from Sinopharm Chemical Reagent.

### Synthesis of Sr_1–*y*_Co_1–*x*_Ir_*x*_O_3−δ_ (SCI, 0 ≤ *x* ≤ 0.2 and 0 ≤ *y* ≤ 0.15)

Typically, the Sr_1–*y*_Co_1–*x*_Ir_*x*_O_3−δ_ (SCI) perovskite samples with different contents of A-site deficiency and Ir incorporation were synthesized by a conventional solid-state method. Powders of stoichiometric amounts of SrCO_3_, Co_3_O_4_, and IrO_2_ were weighed, mixed, and grounded in a mortar for 30 min. The mixture was then transferred to a box furnace (KSL-1200x) and calcined in ambient air at 1100 °C for 12 h. Table 1 shows the denotations of the representative catalysts. The real content of each element was confirmed by SEM–EDX, XPS, and ICP measurements (Table S3-1). The calculated Ir mass content is also shown in Table S3-2.

**Table 1:** Denotation of the Representative Catalysts

composition	denotation
SrCoO_3−δ_	SC
Sr_0.9_Co_0.9_Ir_0.1_O_3−δ_	SCI
Sr_0.9_Co_0.9_Ir_0.1_O_3−δ_ reduced at 350 °C for 3 h	SCI-350

### Synthesis of Sr_0.9_Co_0.9_Ir_0.1_O_3−δ_-T (SCI-T)

The as-obtained samples were then reduced in a tube furnace with a 5% H_2_/N_2_ atmosphere for 3 h at 250, 300, 350, and 400 °C with a ramping rate of 5 °C min^–1^, respectively, to gain a reduction atmosphere-treated electrocatalysts designated as SCI-250, SCI-300, SCI-350, and SCI-400, respectively.

### Material Characterizations

The crystallinity of the materials was characterized by the XRD using an Ultima IV power diffractometer (Rigaku Corporation, Japan) with the Cu Kα radiation (λ = 0.15406 nm). The EXPGUI GSAS software was employed to perform the Rietveld refinement. The Philips-FEI Tecnai F30 TEM (equipped with EDX spectroscopy) was employed to characterize the morphology and fine structure of the material. The HRTEM images were obtained using an FEI Tecnai G2 F20 operated at an accelerating voltage of 200 kV. The elementary dispersity of each sample was measured by using a Zeiss GeminiSEM 500 equipped with an EDX detector and SPECTROBLUE FMX36 ICP–OES instrument, respectively. The chemical state of the materials was analyzed by XPS using a Thermo Scientific K-Alpha instrument (Al Kα radiation = 1486.6 eV). The binding energy of C 1s (284.8 eV) was employed for calibration. The O K-edge X-ray absorption near-edge structure (XANES) were obtained at the Beamlines MCD-A and MCD-B (Soochow Beamline for Energy Materials) at National Synchrotron Radiation Laboratory (NSRL, China). The Ir L_III_-edge XAS data were obtained at the hard X-ray microanalysis (HXMA) beamline, Canadian Light Source. The BET surface areas were measured with the ASAP 2020 instrument and were determined by N_2_ adsorption–desorption isotherms at −196 °C (Figure S34).

### H_2_-TPR Measurement

H_2_-TPR was conducted using the MFTP3060-programmed temperature-pulse adsorption instrument (Xiamen Biotek Co., LTD). First, we inserted an appropriate amount of quartz cotton into the customized quartz tube and placed 100 mg of catalyst into the tube. After the temperature was raised to 850 °C at 10 °C min^−1^ under 5% H_2_/N_2_ gas, it was maintained for another 10 min and then cooled to room temperature. During the measurement, a Wuhao gas chromatography workstation was used to record data.

### In Situ Raman Measurement

In situ Raman testing was performed with a HORIBA’s XploRA PLUS instrument. Before measurements, the catalyst was made into ink and loaded on the glassy carbon electrode in a custom-made electrochemical cell. The glassy carbon electrode, platinum wire, and Ag/AgCl electrode (saturated KCl solution) were used as the working electrode, counter electrode, and reference electrode, respectively. After drying, a 1 M KOH solution was injected into the cell to moisten the catalyst completely. During the test, an electrochemical workstation was used to apply different potentials.

### Dissolution Procedure of Samples for the ICP–OES Test

As the precious metal iridium is more difficult to dissolve, we took 100 mg of catalyst and used a mixture of concentrated nitric acid and concentrated hydrochloric acid (1:20 ratio) to digest the sample. When the solution became clear and transparent, it was transferred to a volumetric flask for constant volume. Then, 15 mL of liquid was taken from the bottle and put into a tube for the ICP–OES test.

### Electrochemical Measurements

To prepare the working electrode, we sonicated a mixture of 10 mg of catalyst, 2 mg of acetylene black, 500 μL of deionized water, 470 μL of absolute ethanol, and 30 μL of 5 wt % Nafion for 1.5 h in an ice bath. 10 μL of uniform ink was dropped onto a rotating disc electrode (RDE) with a diameter of 5 mm, and the electrode completely dried at room temperature. The catalyst loading was 0.51 mg cm^–2^ and the Ir mass loading of the SCI-350/RDE electrode was 48.246 μg cm^–2^ (the Ir content of SCI-350 was determined by ICP–OES).

The OER performance tests were carried out in a traditional three-electrode system at room temperature on a Corrtest 2350H electrochemical station in a PTFE bottle. The RDE, a Hg/HgO electrode, and a Pt wire were used as the working electrode, reference electrode, and counter electrode, respectively. The Hg/HgO electrode was calibrated and its potential was converted to the RHE scale, giving their conversion equation in the 1 M KOH electrolyte: . All electrochemical tests were conducted in 1 M KOH, which was saturated with O_2_ (99.999%) before OER testing. Before the OER measurement, 10 cycles of CV were applied from 1.1 to 1.8 V versus RHE at a scan rate of 100 mV s^–1^ to activate the electrocatalysts. A LSV was measured at a scan rate of 10 mV s^–1^. The Tafel curves were measured at a slow scan rate of 1 mV s^–1^. Meanwhile, all potentials in this work were compensated for iR-drop.

The two-electrode system and the chronopotentiometry (CP) method were used to observe stability in the 1 M KOH solution. To prepare the catalyst electrode, we sonicated a catalyst ink of 10 mg of catalyst, 10 mg of acetylene black, 1 mL of absolute ethanol, and 100 μL of 5 wt % Nafion for 1.5 h in an ice bath. After that, the catalyst ink was dropped on the surface of a carbon cloth, yielding a catalyst mass loading of 1 mg cm^–2^. The catalyst electrode was immersed in a 1 M KOH solution with a dimension of 1 cm × 1 cm. The catalyst was coated on carbon paper for oxygen evolution. For hydrogen evolution, commercial 20 wt % Pt/C was used as the electrode. The stability was also measured with a three-electrode configuration and CP method in 1 M KOH. The catalyst deposited on the carbon paper with a mass loading of 1 mg cm^–2^, a Hg/HgO electrode, and a Pt wire were used as the working electrode, reference electrode, and counter electrode, respectively.

The reference electrode Hg/HgO was calibrated with a RHE in 1 M KOH.^58,59^ First, two Pt electrodes were cycled 45 times in 0.5 M H_2_SO_4_ between −2 and 2 V at a scanning rate of 50 mV s^–1^ for 2 h to clean the surface. Next, the two Pt electrodes were used as the working electrode and counter electrode, respectively. Before calibration, pure H_2_ was bubbled in 1 M KOH for at least 30 min. The LSV curve was performed at a scan rate of 1 mV s^–1^ around the possible zero current potential. The potential of zero net current was the resulting potential. The result showed the potential of zero net was −0.905 V versus the Hg/HgO electrode. Therefore, all the potentials in this work were converted to RHE following the equation
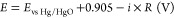
1where *i* was the measured current, *R* (the ohmic resistance of the electrolyte) was obtained by EIS (∼6 Ω in 1 M KOH).

To acquire the ECSA of the electrocatalyst, a series of CVs were measured at different scan rates (20, 40, 60, 80, and 100 mV s^–1^), within the range of potential from 1.1 to 1.2 V versus RHE. By plotting the difference in current density  at 1.15 V vs RHE versus scan rate, the slope can be obtained, which is equal to the double layer capacitance (*C*_dl_).

Then, the ECSA can be determined from eq 2
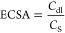
2where *C*_s_ is the specific capacitance of the sample, and typical values fall within the range of *C*_s_ = 22–130 μF cm^–2^ in 1 M KOH. In this work, the value of *C*_s_ is taken as 40 μF/cm^2^.^60^

The MA was normalized with catalyst loading (*m*) from eq 3
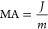
3where *J* (mA cm^–2^) is the current density and *m* is the catalyst loading (0.51 mg cm^–2^).

The SA was normalized with ECSA from eq 4
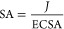
4where *J* (mA cm^–2^) is the current density and the ECSA follows from eq 2.

For pulse voltammetry measurements, the procedure we follow can be found in the previous literature.^33,61,62^ The working electrode was polarized at a specific positive potential (0.5 V vs Hg/HgO) for 30 s, and then switched to the OCP (the average OCP values of SCI and SCI-350 are 0.18 and 0.21 V vs Hg/HgO, respectively.) for another 10 s. The time-dependent current density was recorded, and the cathodic transient current peak was integrated to determine the accumulated positive charge (*Q*). The charge storage capacity (*C*_total_) of the electrocatalyst can be estimated by the slope of (∂*Q*/∂*E*) in the linear region. Then, the pseudocapacitance (*C*_p_) of the catalyst is calculated by the following formula: .

Before the EIS tests, CVs were applied potential between 1.1 and 1.8 V versus RHE at a scan rate of 100 mV s^–1^ to activate the electrocatalysts. The measured potential ranges of the EIS were 1.3–1.7 V versus RHE in the frequency range from 1 × 10^5^ to 1 × 10^–1^ Hz.

The oxygen diffusion coefficient measurements were performed in an Ar saturated 6 M KOH electrolyte with the three-electrode configuration. The detailed procedure could be found in the previous literature.^43^ The RDE, a Hg/HgO electrode, and a Pt wire were used as the working electrode, reference electrode, and counter electrode, respectively. CV was applied from 0.6 to 1.4 V versus RHE at a scan rate of 20 mV s^–1^. To measure the oxygen-ion diffusion coefficient of the catalysts, the chronoamperometry method was employed by applying an additional potential 50 mV more anodic of the *E*_1/2_ (*E*_1/2_ is defined as the potential between pairs of the redox peaks). The potentials applied to SC, SCI, and SCI-350 were 0.1, 0.174, and 0.235 V vs Hg/HgO, respectively, during the chronoamperometry measurements. The rotation rate was applied at 2000 rpm to eliminate the effect of mass transfer. By plotting the current versus the inverse square root of time (*i* vs *t*^–1/2^), the intercept with the *x*-axis (*t*^–1/2^) was based on the fitting of the linear part. According to a bounded three-dimensional diffusion model,^50^ the intercept on the *X*-axis was used to calculate the oxygen diffusion coefficient, using eq 5 as follows
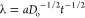
5where λ is a dimensionless shape factor of 2. *D*_o_ is the oxygen diffusion coefficient. *a* can be based on following eq 6
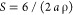
6where *S* is the BET area of the catalysts (Table S6) and the ρ is theoretical density of the material.

The DEMS tests were performed using a QAS 100 mass spectrometer with a detector (Linglu Instruments, Shanghai). To prepare the catalyst ink, we sonicated a mixture of 5 mg of catalyst, 500 μL of deionized water, 460 μL of absolute ethanol, and 40 μL of 5 wt % Nafion for 1.5 h in an ice bath. Before measurements, 30 μL of uniform ink was dropped onto the gold film and replaced several times in the 1 M KOH solution with H_2_^18^O. Then, the SCI-350 labeled with ^18^O was used as a working electrode. The Ag/AgCl electrode with the saturated KCl solution and a Pt wire acted as the reference electrode and counter electrode, respectively. The Ag/AgCl electrode (saturated KCl solution) was converted to the RHE, giving their conversion equation in the 1 M KOH electrolyte: . The CVs were applied from 0 to 0.8 V versus Ag/AgCl (saturated KCl solution) at a scan rate of 5 mV s^–1^ in the 1 M KOH solution with H_2_^16^O. At the same time, gas products were monitored with different molecular weights by mass spectrometry.

### Zero-Gap Alkaline Water Electrolyzer Assembly

Electrochemical measurements were performed with a CHI 660E electrochemical station (Chenhua, Shanghai) at room temperature. A self-supporting two-electrode alkaline water electrolyzer was fabricated using SCI-350/NF and CoP/NF as the anode and cathode, respectively. To assemble the SCI-350/NF electrode, a catalyst ink dispersing 6 mg of SCI-350 powder and 2 mg of Vulcan XC-72 carbon in 1 mL of a mixed solution containing 950 μL of absolute ethanol and 50 μL of Nafion solution (5 wt %) was prepared. In addition, catalyst ink was dropped on a piece of nickel foam surface, yielding a catalyst mass loading of 1 mg cm^–2^. To prepare the C/NF electrode, the ink without the catalyst was dropped onto a piece of nickel foam surface, resulting in a mass loading of 1 mg cm^–2^. Two electrodes, separated by an alkaline battery membrane (YLD-GS, Xinxiang Heluelida Power Sources Co. Ltd), were immersed in a 6 M KOH solution with a dimension of 0.5 cm × 0.5 cm. The test temperature was controlled at 25 °C. LSV was recorded from 0 to 2.1 V with a scan rate of 10 mV s^–1^. The EIS measurement was performed at an OCP in the frequency range of 10^5^ to 10^–2^ Hz. The polarization curve was iR corrected to compensate for the effect of solution resistance. To assess the long-term stability, CP tests were carried out at a constant current density of 500 mA cm^–2^.

### Theoretical Calculations

The first-principles^63,64^ were employed to perform all (spin-polarization DFT) calculations within the generalized gradient approximation (GGA) using the Perdew–Burke–Ernzerhof (PBE)^65^ formulation. The projected augmented wave (PAW) potentials^66,67^ were selected to describe the ionic cores and take valence electrons into account using a plane wave basis set. The kinetic energy cutoff is 450 eV. Partial occupancies of the Kohn–Sham orbitals were allowed using the Gaussian smearing method and a width of 0.05 eV. The electronic energy was regarded as self-consistent while the energy change was <10^–4^ eV. A geometry optimization was acknowledged as convergent while the energy change was <0.05 eV Å^–1^. The *U* correction is employed for Ir and Co atoms in our material systems. The vacuum spacing perpendicular to the plane of the slab is set to 20 Å. In our work, the supercell Sr_2_Co_2_O_6_(110) surface had been established from the Sr_2_Co_2_O_6_ crystal structure. And the Ir is randomly doped on the structure Sr_2_Co_2_O_6_(110) surface to form a stable structure, where the lattice parameter is *a* = 6.893 Å, *b* = 17.137 Å, and *c* = 43.1213 Å. For interface structures, the Sr_2_Co_2_O_6_(110)/CoOOH–IrO_2_ structures had been established with the CoOOH(2000) and IrO_2_ (101) structures to form a stable interface structures. In our structure, the *U* correction is used for Ir(4.36 eV) and Co(3.95 eV) atoms. For the irreducible Brillouin zone sampling, a Monkhorst–Pack *k*-point grid of 3 × 3 × 1 was adopted for all models. For the calculation of Gibbs free energy during OER, the adsorption energies (*E*_ads_) were calculated using the following equation: , where *E*_ad/mat_, *E*_ad_, and *E*_mat_ are referred to the total energies of the optimized adsorbate/material system, the adsorbate in the structure, and the clean material, respectively. The free energy was calculated using eq 7
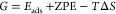
7where *G*, *E*_ads_, ZPE, and *T*Δ*S* are the free energy, total energy from DFT calculations, zero-point energy, and entropic contributions, respectively. Finally, the reaction energies (*G*) of different intermediates are defined as Δ*G* = *G*_i_ – *R*_eactant_ (*G*_i_ is the energy of intermediates and *R*_eactant_ is the total energy of the reactants). The reaction free energies (DG) for each step in both AEM and LOM pathways were calculated, and the step with the highest energy barrier was identified as the RDS.

## Supporting Information Available

XRD, TEM, XPS, double-layer capacitance (*C*_dl_), in situ electrochemical impedance spectroscopy plots, additional electrochemical data, EXAFS spectra, HAADF-STEM, EDX, and TEM mapping, LSV curves, Tafel slope, H_2_-TPR profiles, normalized mass activity, CV, PDOS, EXAFS, fitting results, comparison of OER performance, BET surface area, oxygen diffusion coefficient, and comparison of AWE performance (PDF)
