# Supplementary material for: Ex Situ Reconstruction-Shaped Ir/CoO/Perovskite Heterojunction for Boosted Water Oxidation Reaction
Source: ACS Catal. 2023 Mar 29;13(7):5007–19. doi: 10.1021/acscatal.2c05684 (PMC10088023; doi:10.1021/acscatal.2c05684)
Supplement: Supplementary file 1 — cs2c05684_si_001.pdf [file cs2c05684_si_001.pdf]

Supporting Information

Ex-situ Reconstruction Shaped Ir/CoO/Perovskite Heterojunction for Boosted Water Oxidation Reaction

Hongquan Guo[a,‡], Yanling Yang[a,‡], Guangming Yang[b], Xiaojuan Cao[c], Ning Yan[c], Zhishan Li[a], Emily Chen[d], Lina Tang[e], Meilan Peng[a], Lei Shi[f], Shunji Xie[g,h], Huabing Tao[g,h], Chao Xu[i], Yinlong Zhu[j], Xianzhu Fu[k], Yuanming Pan[l], Ning Chen[m], Jinru Lin[n], Xin Tu[i,*], Zongping Shao[b] and Yifei Sun[a,g,o,*]

[a] College of Energy, Xiamen University, Xiamen 361005, China.
[b] State Key Laboratory of Materials-Oriented Chemical Engineering, College of Chemical Engineering, Nanjing Tech University, Nanjing, 211816, China.
[c] School of Physics and Technology, Wuhan University, Wuhan 430072, China.
[d] Monash Centre for Electron Microscopy, Monash University, Victoria 3800, Australia.
[e] Key Laboratory of Low-grade Energy Utilization Technologies and Systems, MOE, Chongqing University, Chongqing 40030, China.
[f] School of Chemical Engineering, Dalian University of Technology, Dalian 116024, China.
[g] State Key Laboratory of Physical Chemistry of Solid Surface, Xiamen University, Xiamen 361005, China.
[h] College of Chemistry and Chemical Engineering, Xiamen University, Xiamen 361005, China.
[i] Department of Electrical Engineering and Electronics, University of Liverpool, Liverpool, Liverpool L69 3GJ, UK.
[j] Institute for Frontier Science, Nanjing University of Aeronautics and Astronautics, Nanjing 210001, China.
[k] Shenzhen Institutes of Advanced Technology, Chinese Academy of Sciences, Shenzhen 518055, China.
[l] Department of Geological Sciences, University of Saskatchewan, Saskatoon, SK S7N 5E2, Canada.
[m] Canadian Light Source, University of Saskatchewan, Saskatoon, SK S7N 0X4, Canada.
[n] Key Laboratory of Pollution Ecology and Environmental Engineering, Institute of Applied Ecology, Chinese Academy of Sciences, Shenyang, Liaoning 110016, China.
[o] Shenzhen Research Institute of Xiamen University, Shenzhen, 518057, China.

Correspondence Email: xin.tu@liverpool.ac.uk (X. Tu); yfsun@xmu.edu.cn (Y.F. Sun)
[‡]These authors contributed equally: Hongquan Guo, Yanling Yang.

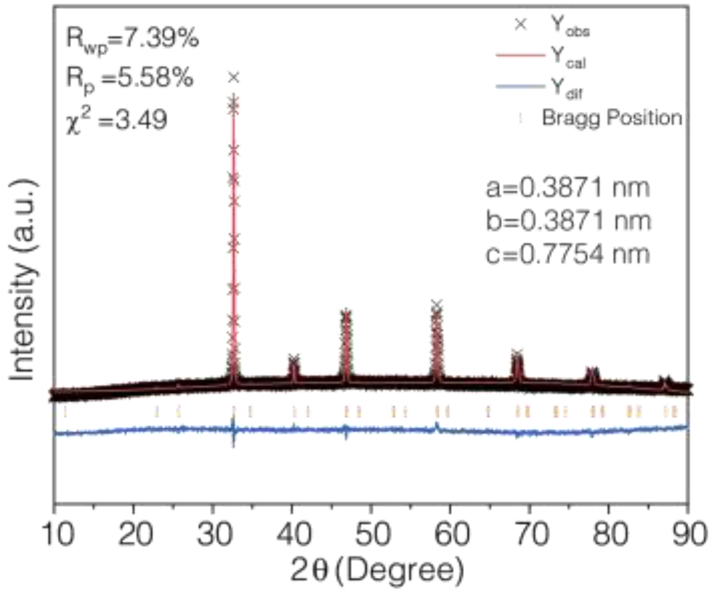

Figure S1. Rietveld refinement of X-ray diffraction (XRD) patterns of tetragonal SCI electrocatalyst. The detailed lattice parameters are shown in the Figure.

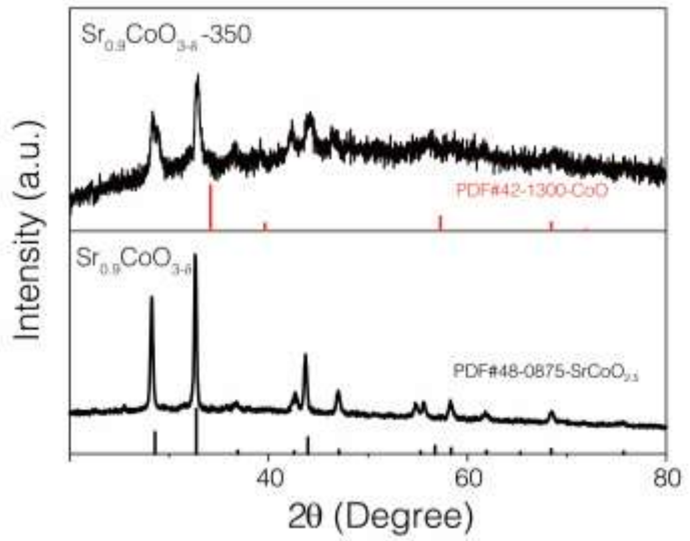

Figure S2. The XRD pattern of $Sr_{0.9}CoO_{3-\delta}$ and $Sr_{0.9}CoO_{3-\delta}$ reduced at 350 ºC for 3 hours. After reduction, the diffraction peaks of perovskite become less intensive and broader, indicative of the weakened crystallinity. However, the diffraction peaks ascribed to CoO are still not detectable (red lines), suggesting the maintenance of perovskite without phase separation.

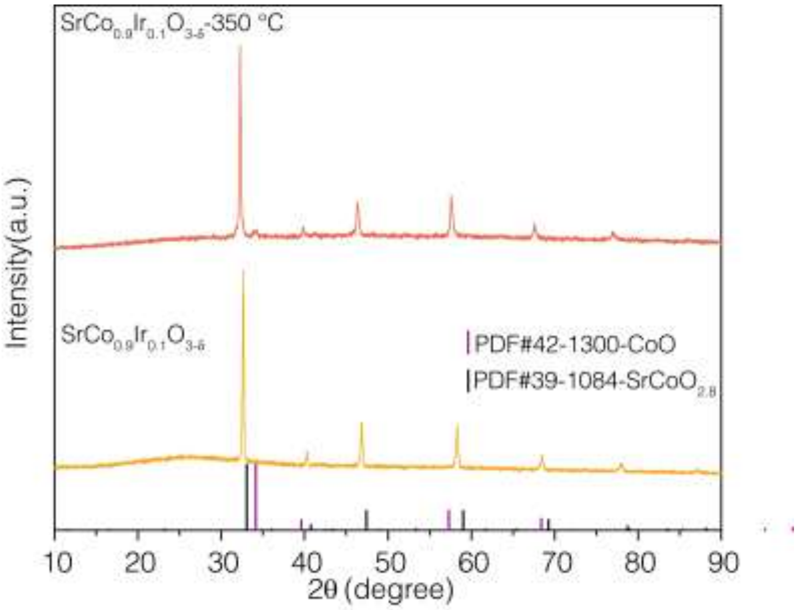

Figure S3. The XRD patterns of A-site deficiency free $SrCo_{0.9}Ir_{0.1}O_{3-\delta}$ and $SrCo_{0.9}Ir_{0.1}O_{3-\delta}$ reduced at 350 °C for 3 hours. The (111) plane of CoO can be detected on reduced electrocatalyst, illustrating that the reduction treatment leads to the phase separation and the segregation of B-site cations. The results in Figures S2-S3, Supporting Information, collectively confirmed that the Ir incorporation could facilitate the formation of CoO while the materials were exposed to reducing atmosphere.

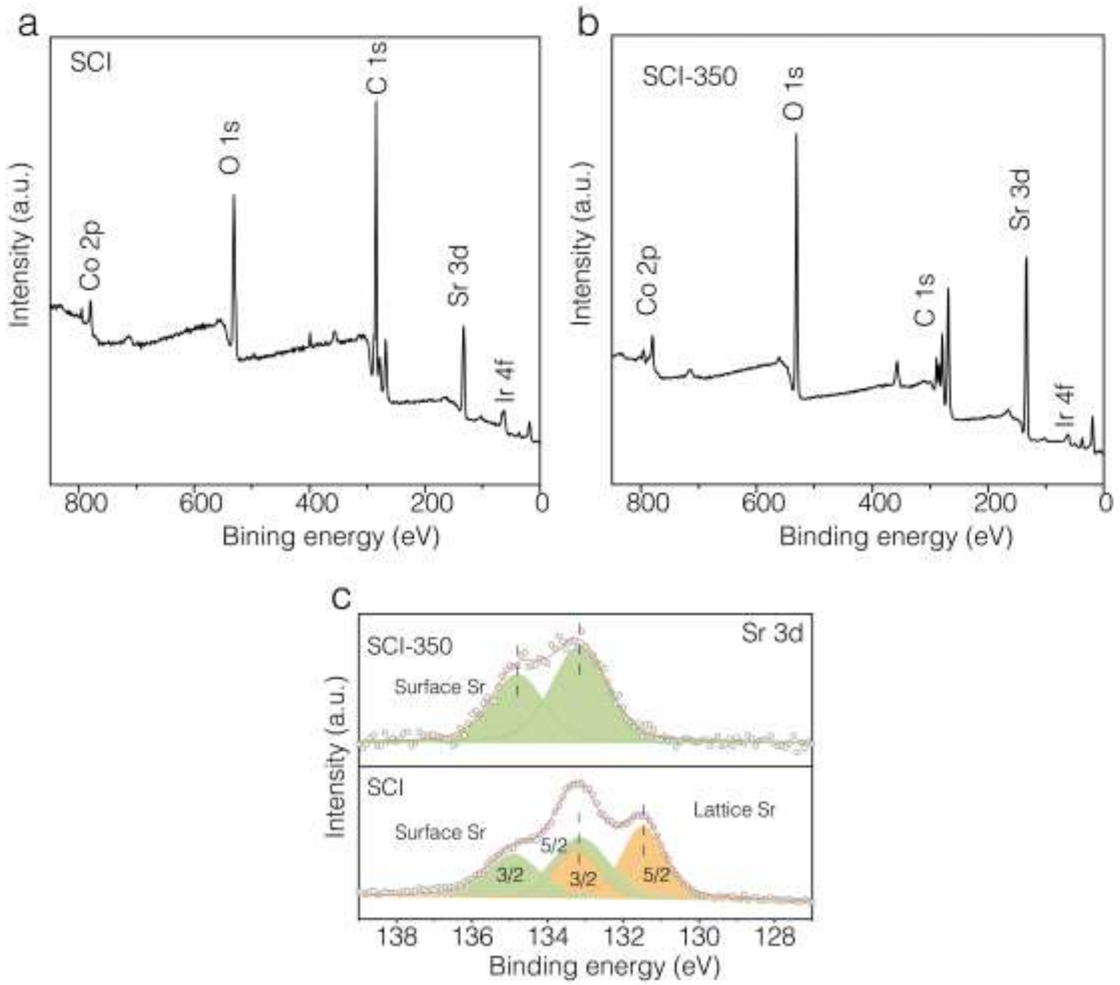

Figure S4. XPS survey spectra of (a) SCI and (b) SCI-350. (c) XPS spectra of Sr 3d of SCI and SCI-350.

The Sr 3d XPS spectra of Sr-containing perovskites were well known to be sensitive to the surface structural rearrangement. The Sr 3d signal from SCI can be fitted with two doublets. The first doublet marked in yellow should be related to the Sr $3d_{5/2}/3d_{3/2}$ peaks from the perovskite lattice, with binding energy peak locations of approximately 131.4 eV and 133.2 eV, respectively. The second doublet (shown in green), having the Sr $3d_{5/2}/3d_{3/2}$ peaks at approximately 133.1 eV and 135.1 eV, can be assigned to surface Sr.[1, 2]. After reduction, it is shown that the first doublet almost disappears on SCI-350, and the position and intensity of the second doublet are maintained well. These results are consistent with the characterization results in Figure 3, revealing that SCI-350 undergoes surface reconstruction but shows no detectable cation loss after reduction.

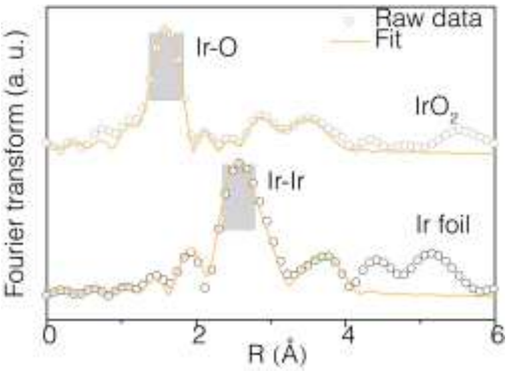

Figure S5. k^3-weighted Ir L_{III}-edge EXAFS spectra of IrO_2 and Ir**.**

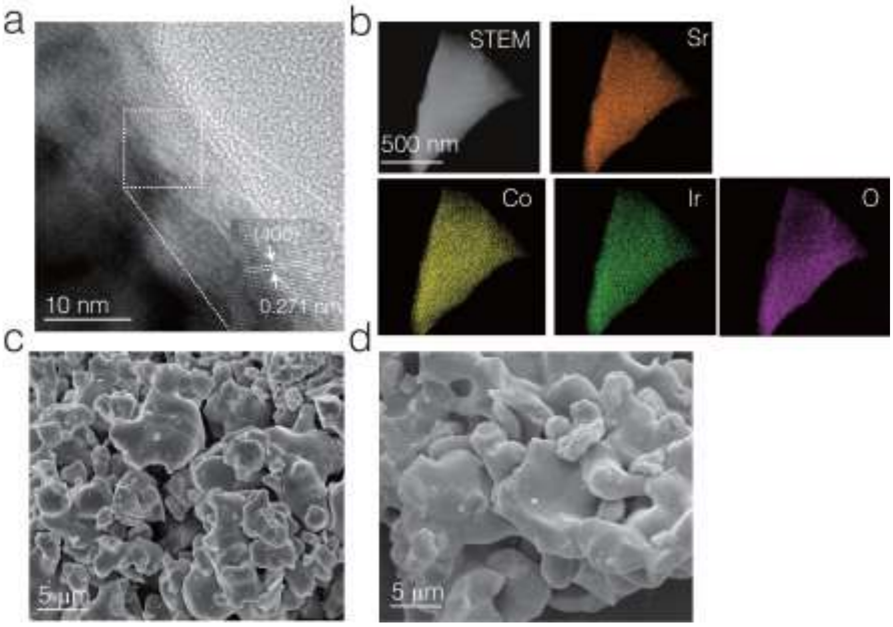

Figure S6. (a) TEM, (b) HAADF-STEM and EDX mapping results of SCI. The d-spacing of 0.271 nm can be ascribed to the (400) plane of SCI (the PDF card number is PDF#39-1084). SEM images of (c) SCI and (d) SCI-350. The EDX mapping results prove the uniform distribution of all elements.

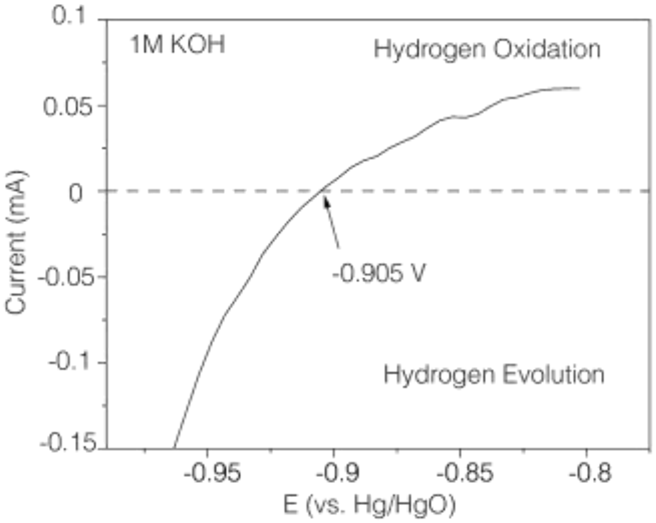

Figure S7. The calibration plot of Hg/HgO reference electrode. The potential of reversible hydrogen electrode is -0.905 V vs. Hg/HgO.

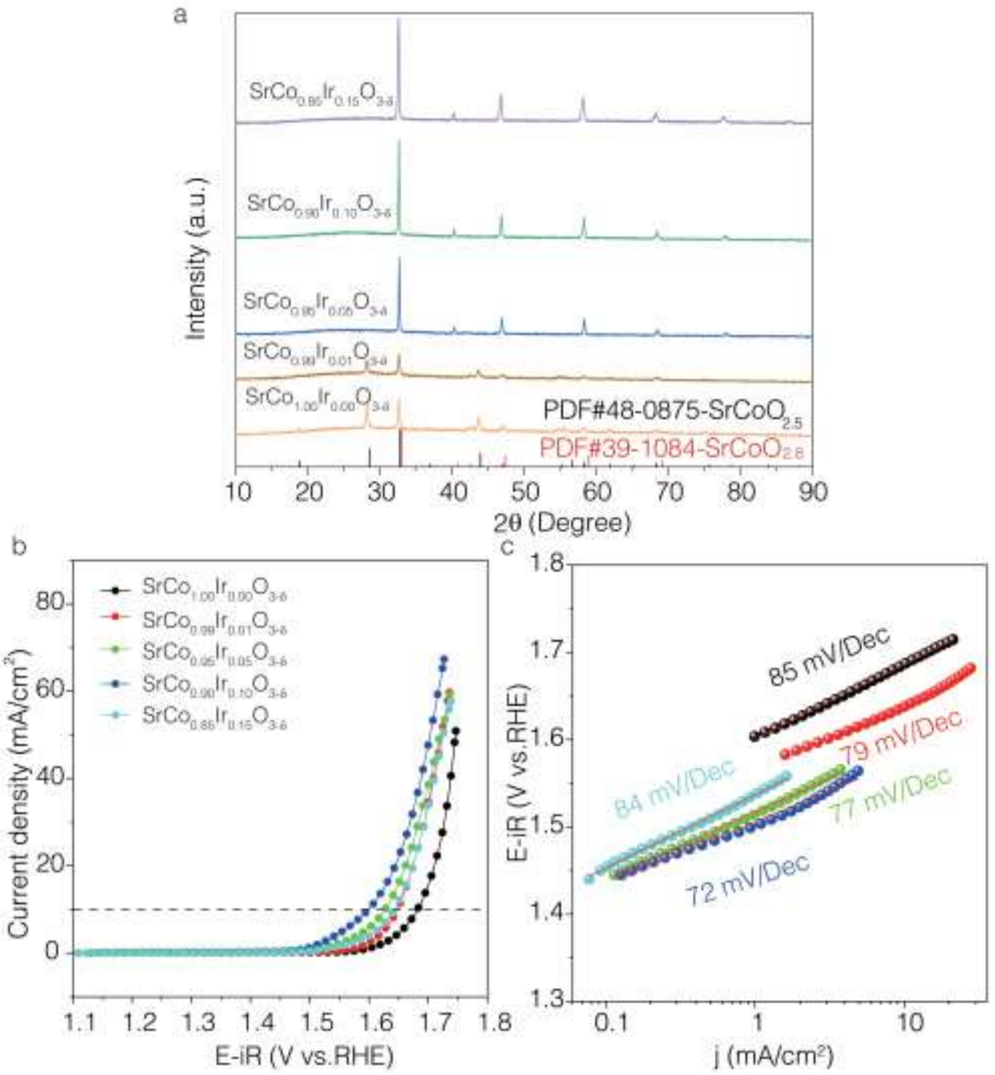

Figure S8. The screen of Ir content. (a) The XRD pattern, (b) The LSV curves, and (c) The Tafel slope of $SrCo_{1-x}Ir_xO_{3-\delta}$ electrocatalysts of various Ir content (X=0~0.15). The molar content of Ir is optimized to be 0.1 with the lowest overpotential of 370 mV (at 10 mA cm^{-2}) and Tafel slope of 77.4 mV Dec^{-1}.

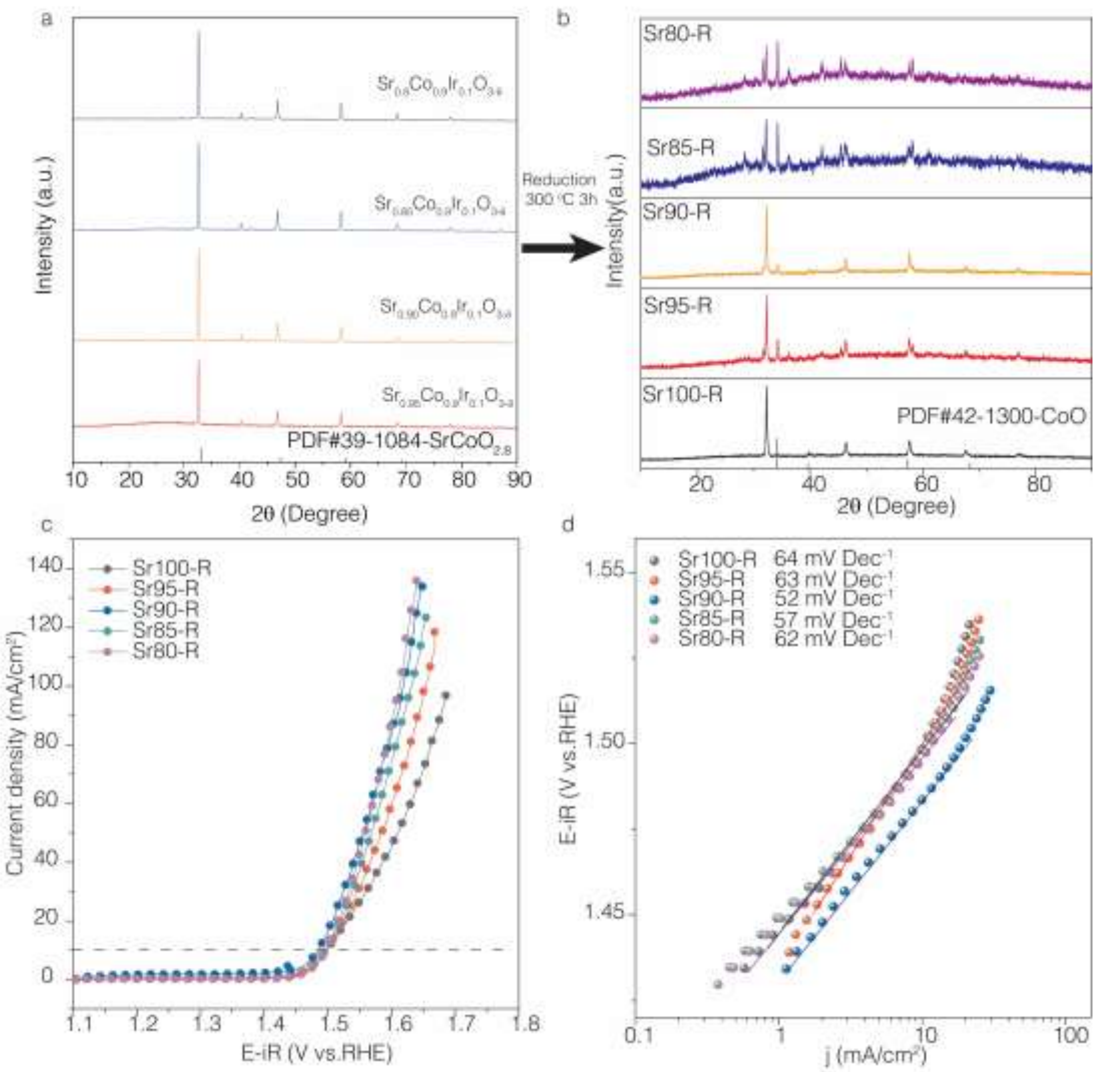

Figure S9. The screen of Sr content (A-site deficiency). (a) The XRD pattern of $Sr_{1-x}Co_{0.9}Ir_{0.1}O_{3-\delta}$ electrocatalysts of various Sr content (X=0~0.2). (b) The XRD pattern. (c) The LSV curves, and (d) The Tafel slope of $Sr_{1-x}Co_{0.9}Ir_{0.1}O_{3-\delta}$ electrocatalysts reduced at 300 ºC for 3 hours. The introduction of A-site deficiency strengthens the segregation of CoO. The content of Sr is optimized to be 0.9 with the lowest overpotential of 255 mV (at 10 mA cm^{-2}) and Tafel slope of 52 mV Dec^{-1}.

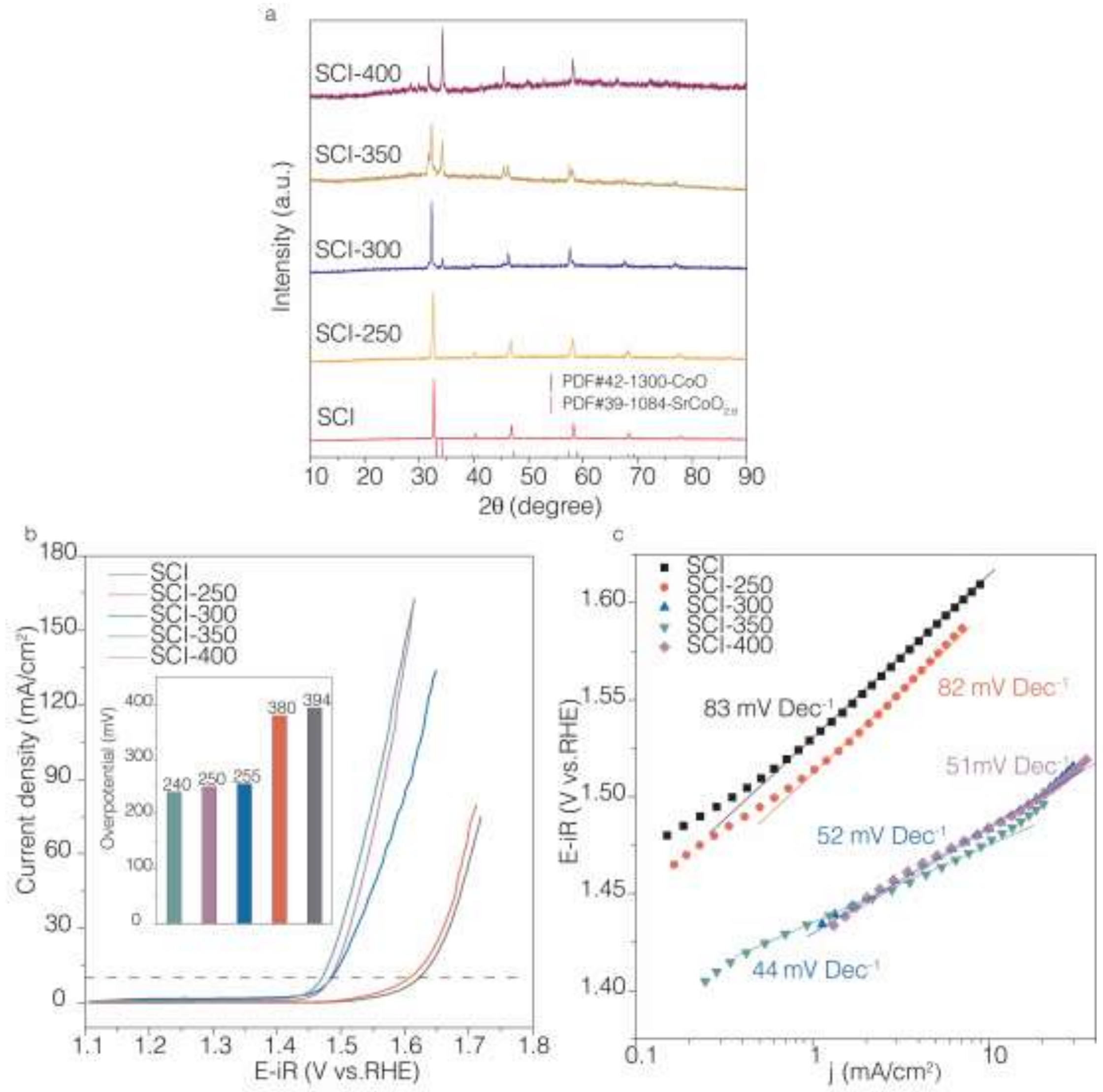

Figure S10. The screen of reduction temperature. (a) The XRD pattern of, (b) The LSV curves, and (c) The Tafel slope of $Sr_{0.9}Co_{0.9}Ir_{0.1}O_{3-\delta}$ electrocatalysts reduced at different temperatures (250 ~ 400 ºC) for 3 hours. Higher temperature leads to the larger content of CoO. The temperature optimized to be 350 ºC with the lowest overpotential of 240 mV (at 10 mA cm^{-2}) and Tafel slope of 44 mV Dec^{-1}.

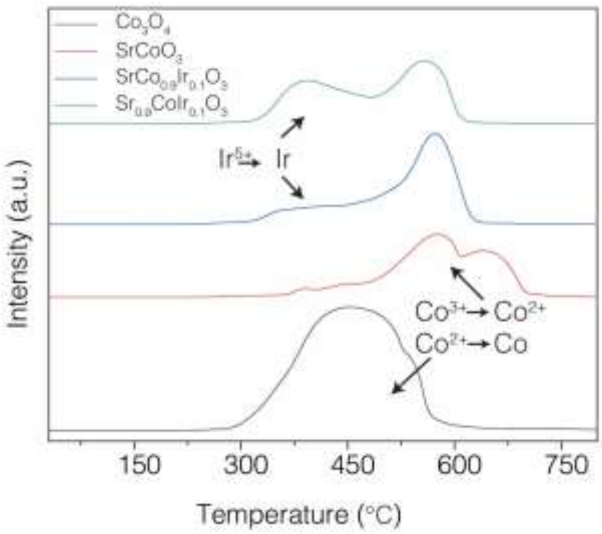

Figure S11. The H_2-TPR (hydrogen-temperature programmed reduction) profiles of various electrocatalysts. The Co_3O_4 shows a broad reduction peak ranging from 300 to 600 ºC in which the multi-step reduction including Co^{3+} to Co^{2+} and Co^{2+} to Co can be expected.[3] Differently, the Co cations in $SrCoO_{3-\delta}$ exhibit completely different reduction behavior with the reduction peak at higher temperature regions between 500 and 700 ºC. On the other hand, the reduction of oxidized Ir to metallic Ir can be observed at the temperature of ~350 ºC in the curve of SCI. The introduction of A-site deficiency facilitates the reduction of Ir by intensifying its reduction peak at the temperature region of 350 ºC.

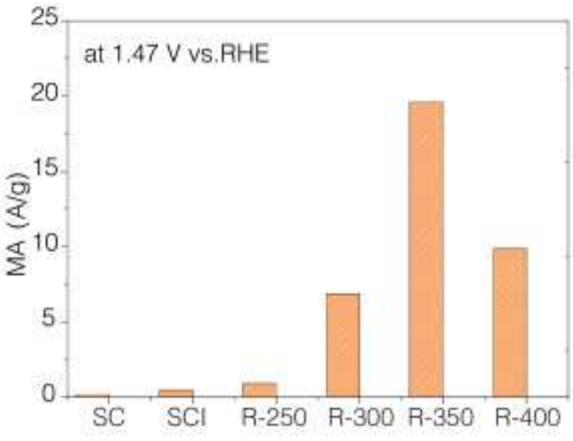

Figure S12. The normalized mass activity of various electrocatalysts.

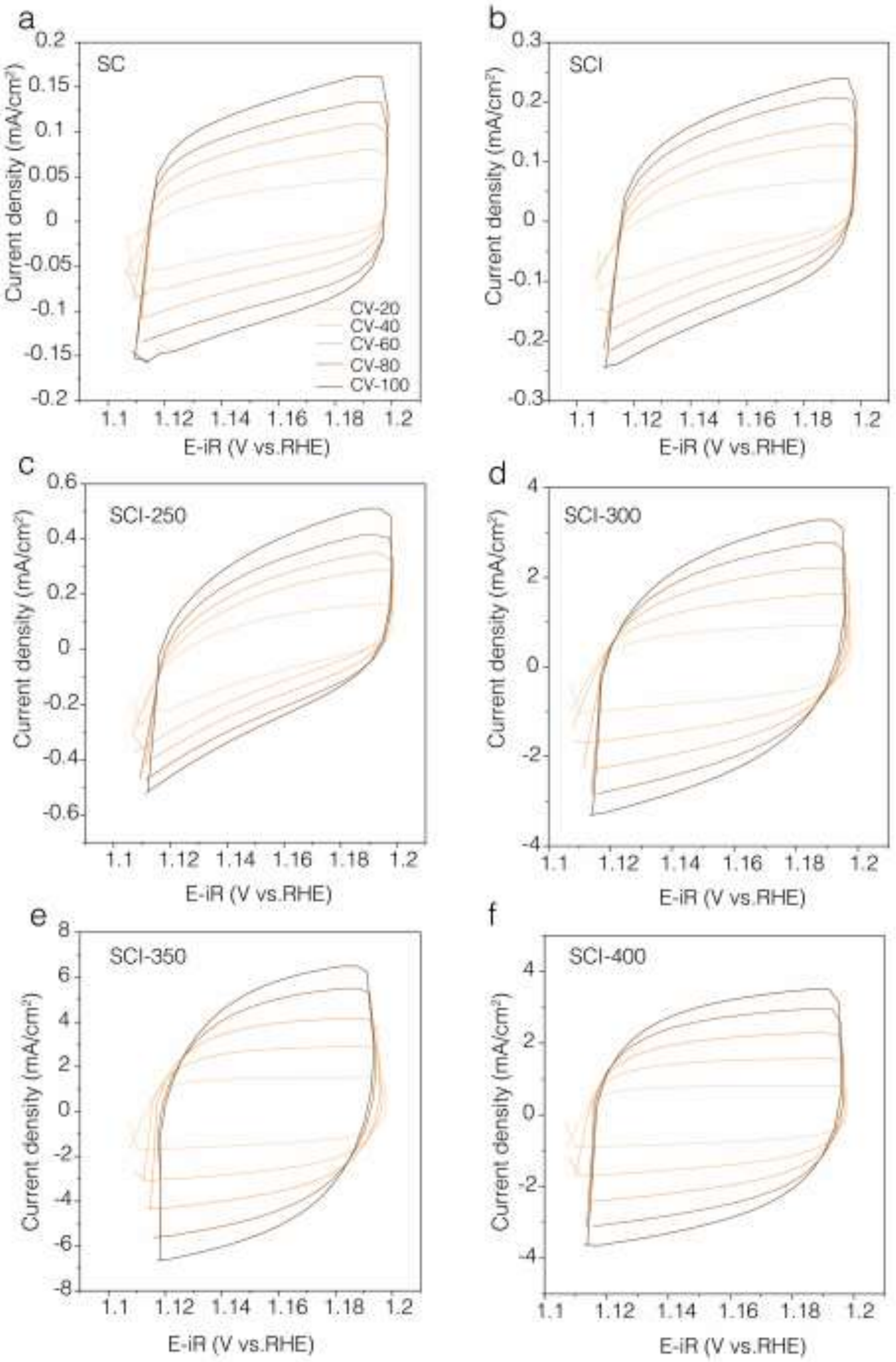

Figure S13. (a-f) Cyclic voltammograms (CV) of different catalysts at various scan rates from 20 mV s^{-1} to 100 mV s^{-1} within the potential range of 1.1 ~1.2 V vs. RHE.

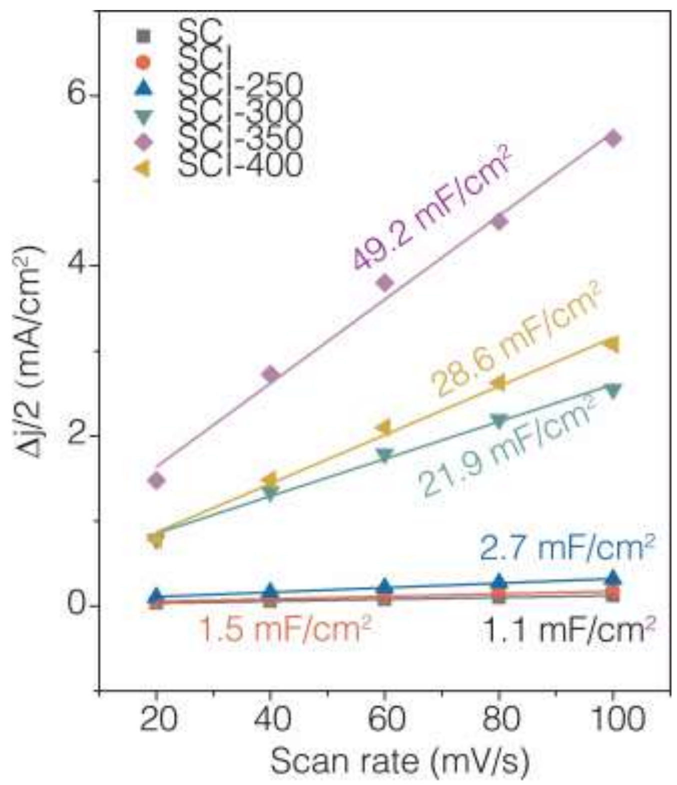

Figure S14. The Δj/2 vs. scan rate plot of different samples.

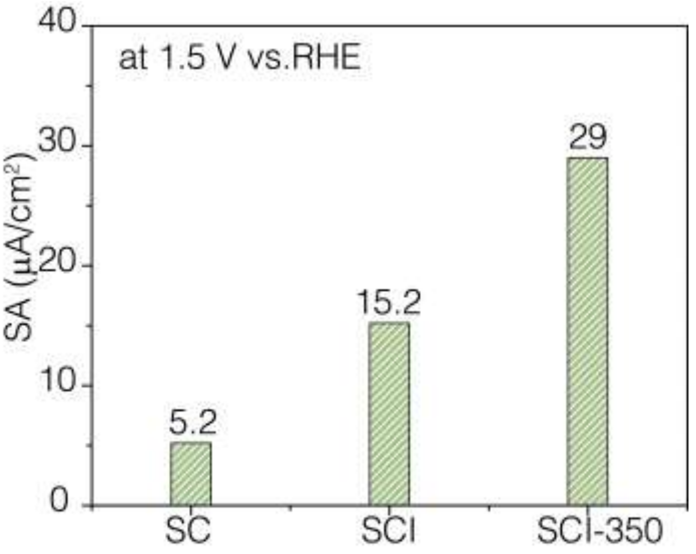

Figure S15. The specific ECSA activity of various catalysts at 1.5 V.

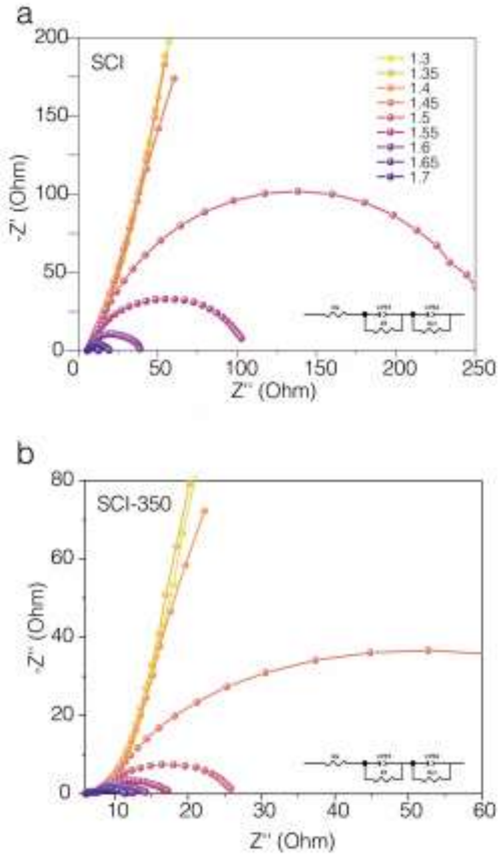

Figure S16. Nyquist plots for (a) SCI and (b) SCI-350 catalysts at different applied potentials (from 1.3 V to 1.7V) versus RHE in 1 M KOH.

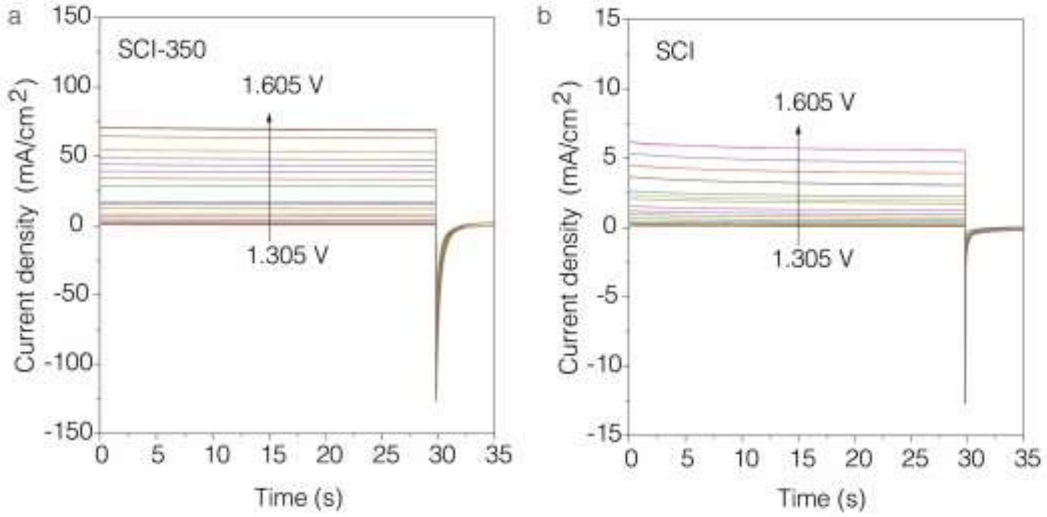

Figure S17. Representative pulse voltammetry protocol of SCI and SCI-350 electrocatalysts. The plots demonstrate the potential step from 1.305 V to 1.605 V, and the corresponding pulse current responses.

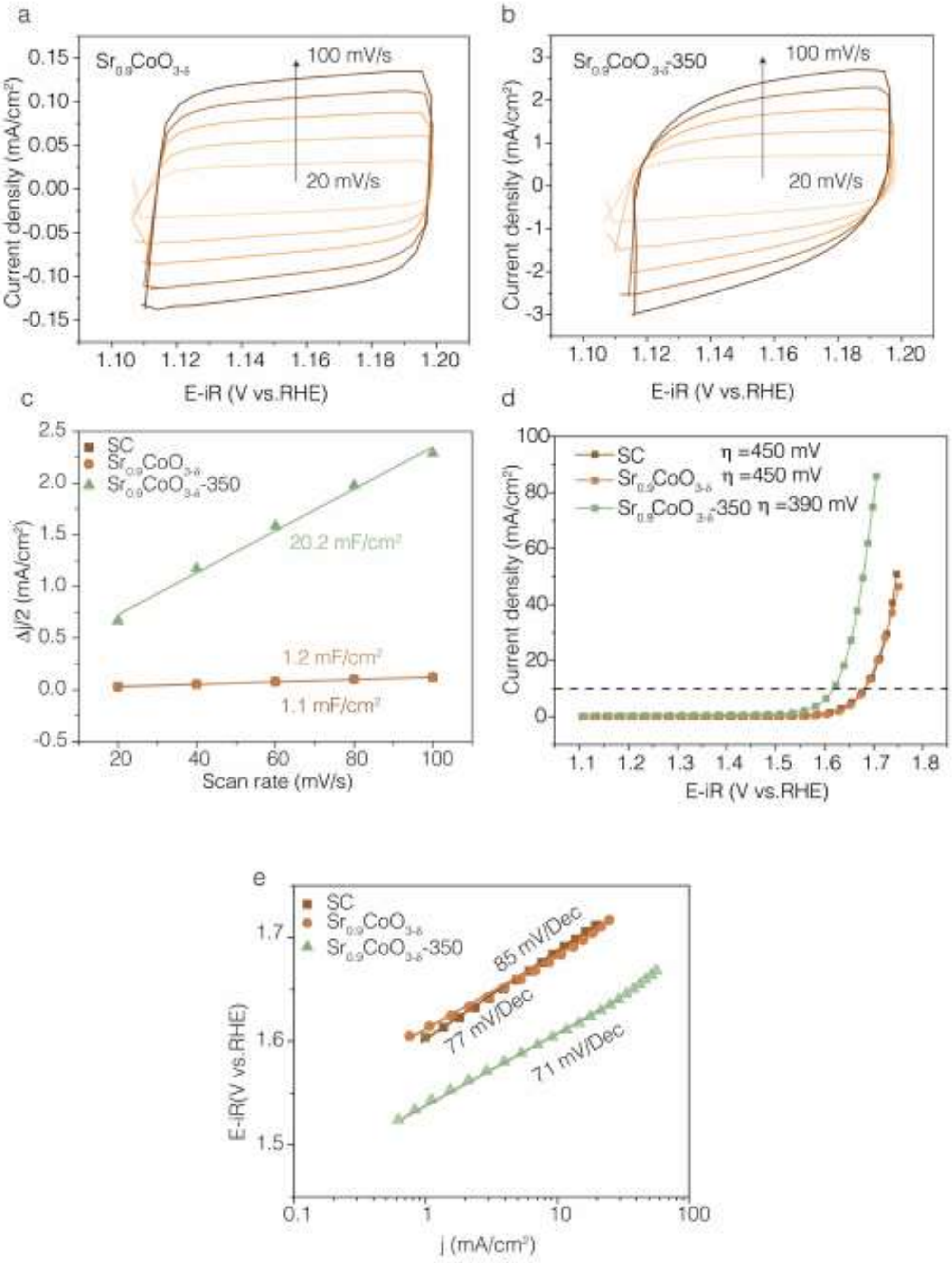

Figure S18. The evaluation of reduced/pristine $Sr_{0.9}CoO_{3-\delta}$ electrocatalyst. The reduced $Sr_{0.9}CoO_{3-\delta}$ refers to $Sr_{0.9}CoO_{3-\delta}$ reduced at 350 ºC for 3 hours in 5% H_2/N_2. Cyclic voltammograms (CV) of (a) $Sr_{0.9}CoO_{3-\delta}$ and (b) reduced $Sr_{0.9}CoO_{3-\delta}$. (c) The Δj/2 vs. scan rate plot. The (d) LSV and (e) Tafel plot of various electrocatalysts.

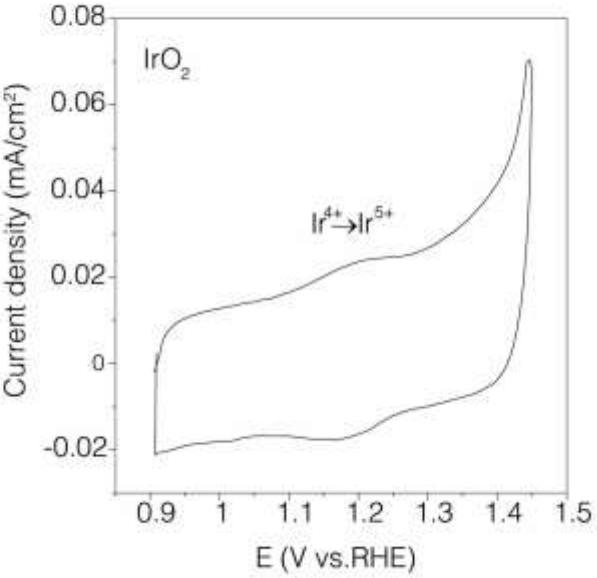

Figure S19. Cyclic voltammograms (CV) of IrO_2 electrocatalyst. The redox peak at around 1.2 V vs. RHE can be observed, which can be ascribed to Ir^{4+}/Ir^{5+} pair.[4]

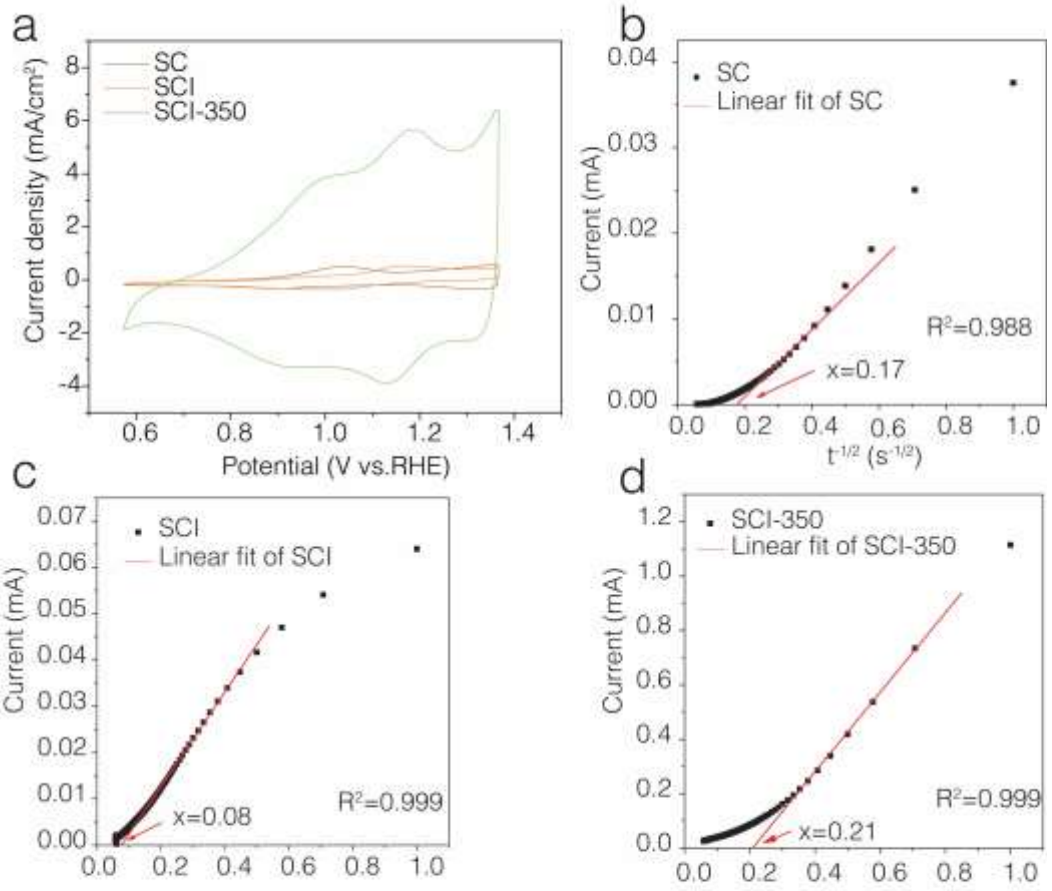

Figure S20. (a) CV curves of SC, SCI and SCI-350 in Ar-saturated 6 M KOH. Oxygen diffusion coefficient experiments of (b) SC, (c) SCI and (d) SCI-350.The redox peaks are correlated to the insertion of oxygen containing species into and their extraction from the accessible lattice vacancy sites. SCI-350 clearly displays a higher redox current density, indicating its capability for oxygen intercalation.

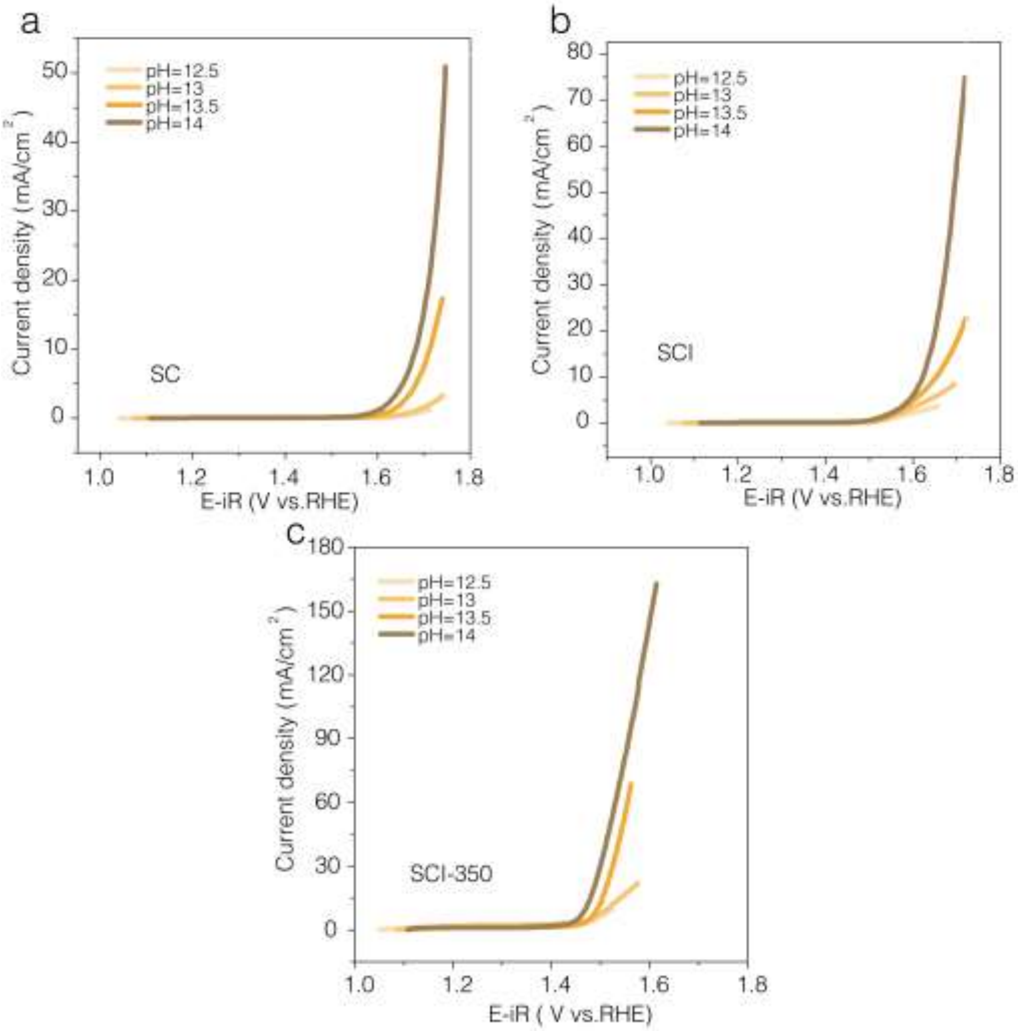

Figure S21. pH dependence (pH =12.5-14) of the OER activities of (a) SC, (b) SCI, and (c) SCI-350.

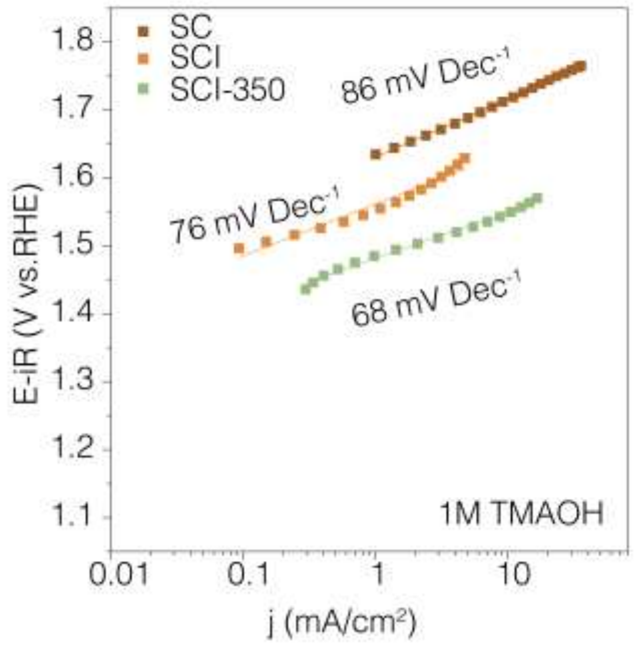

Figure S22. The Tafel slope of various electrocatalysts in 1M TMAOH electrolyte.

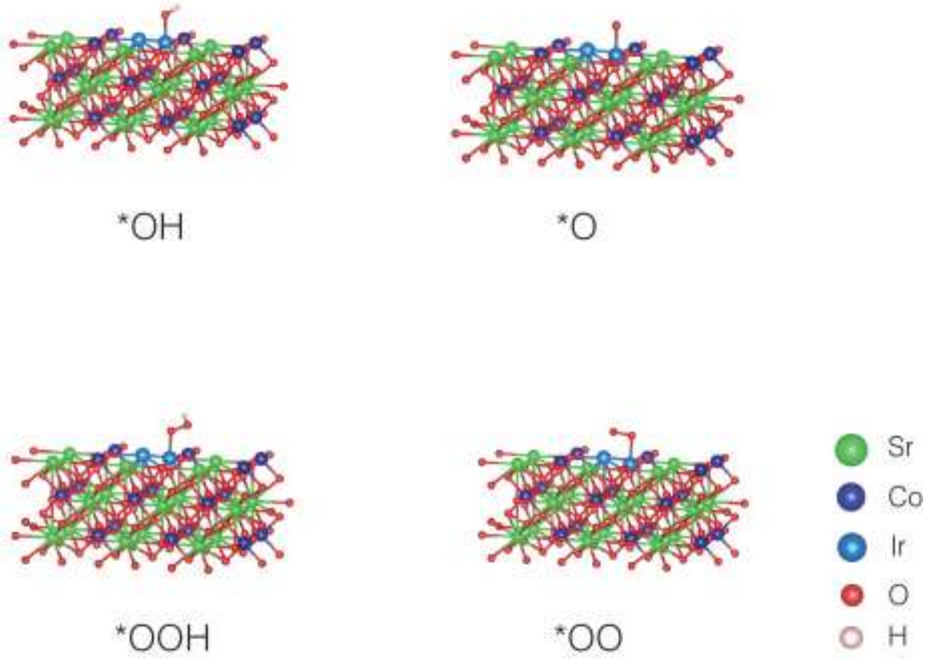

Figure S23. The schematic OER pathway for SCI via AEM. The green, purple, blue, red, and pink spheres represent Sr, Co, Ir, O, and H atoms, respectively.

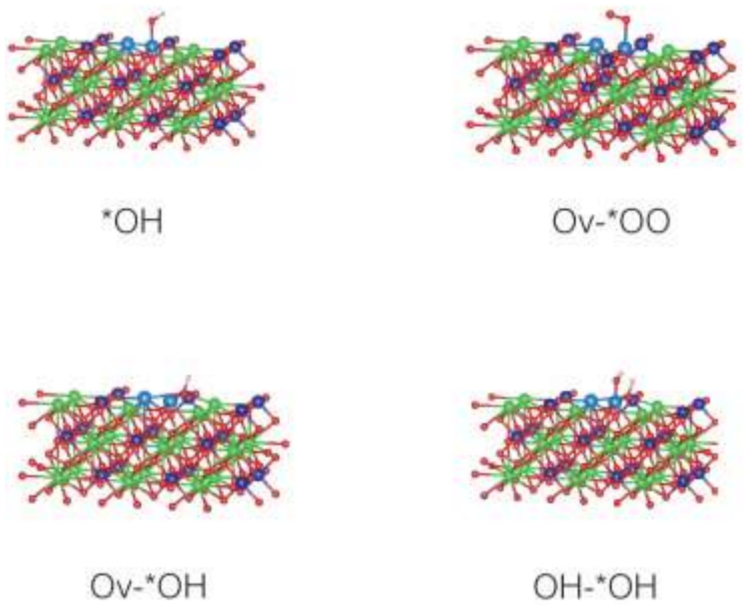

Figure S24. The schematic OER pathway for SCI via LOM.

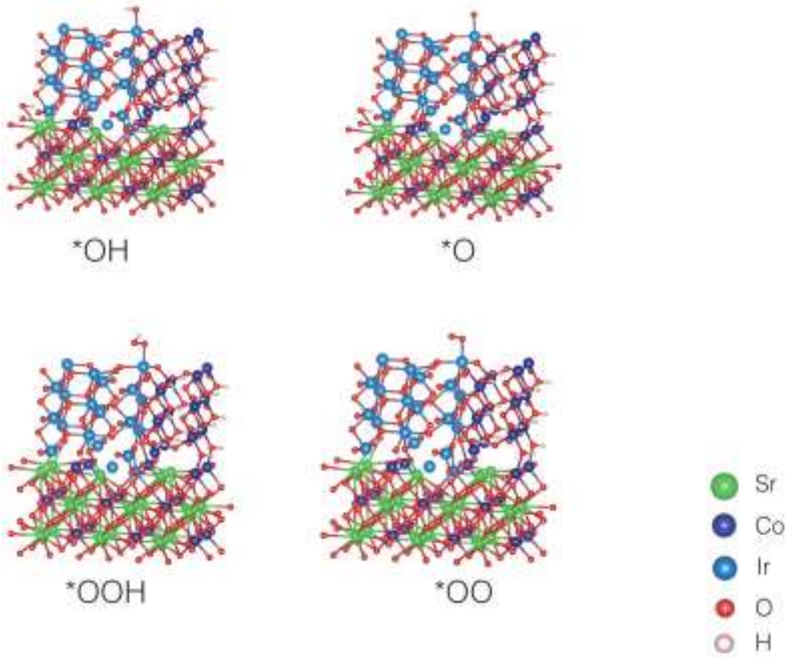

Figure S25. The schematic OER pathway for SCI-350 via AEM.

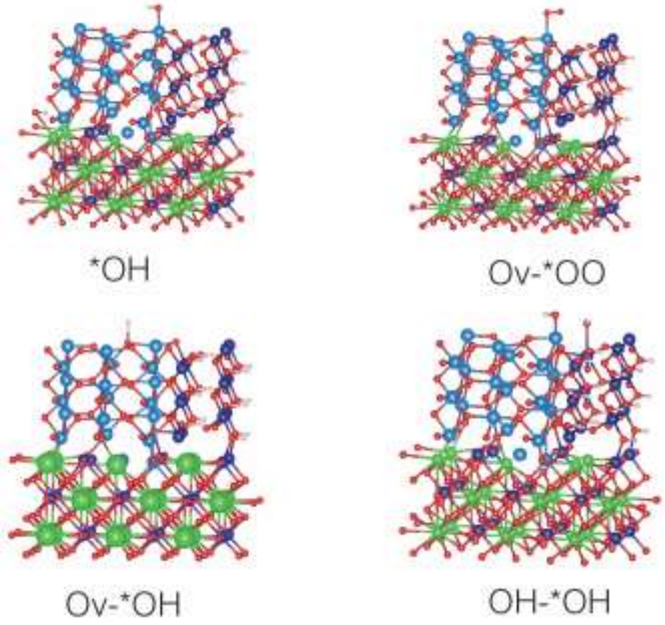

Figure S26. The schematic OER pathway for SCI-350 via LOM.

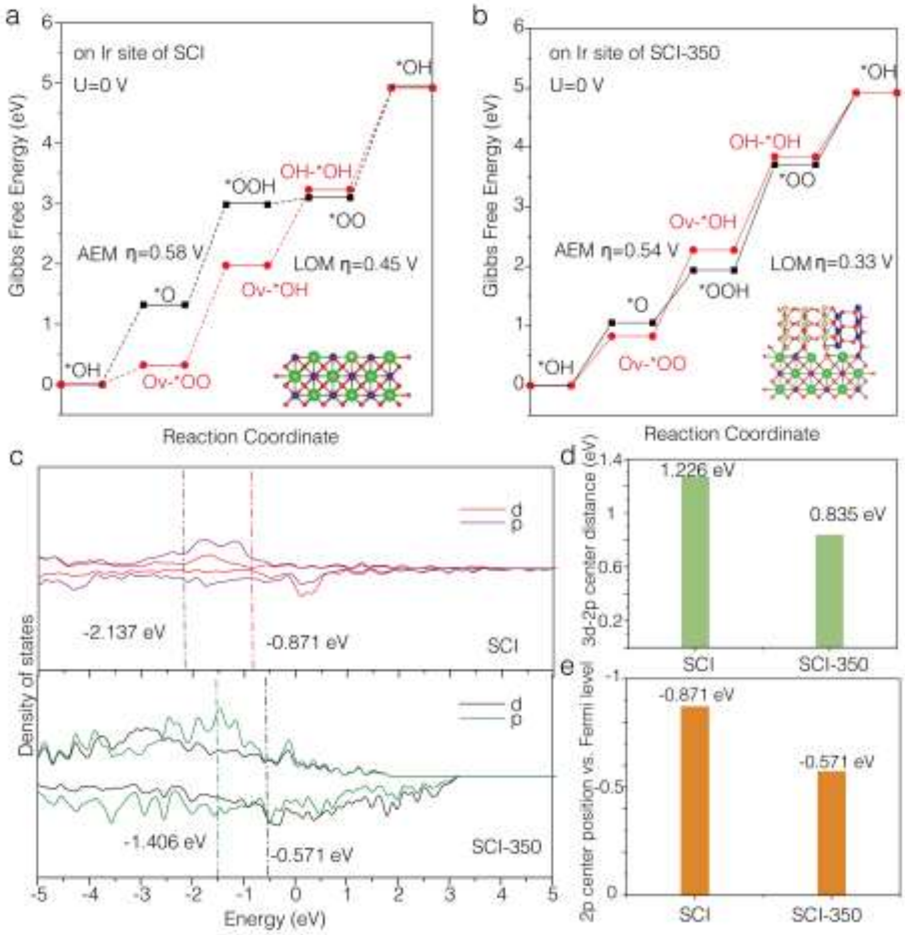

Figure S27. (a-b) The Gibbs free energy diagram of AEM and LOM pathways on SCI and SCI-350. (c) Computed partial electronic density of states (PDOS) of SCI and SCI-350. (d) The metal 3d orbital - O 2p orbital distance and (e) O 2p center position plots of SCI and SCI-350.

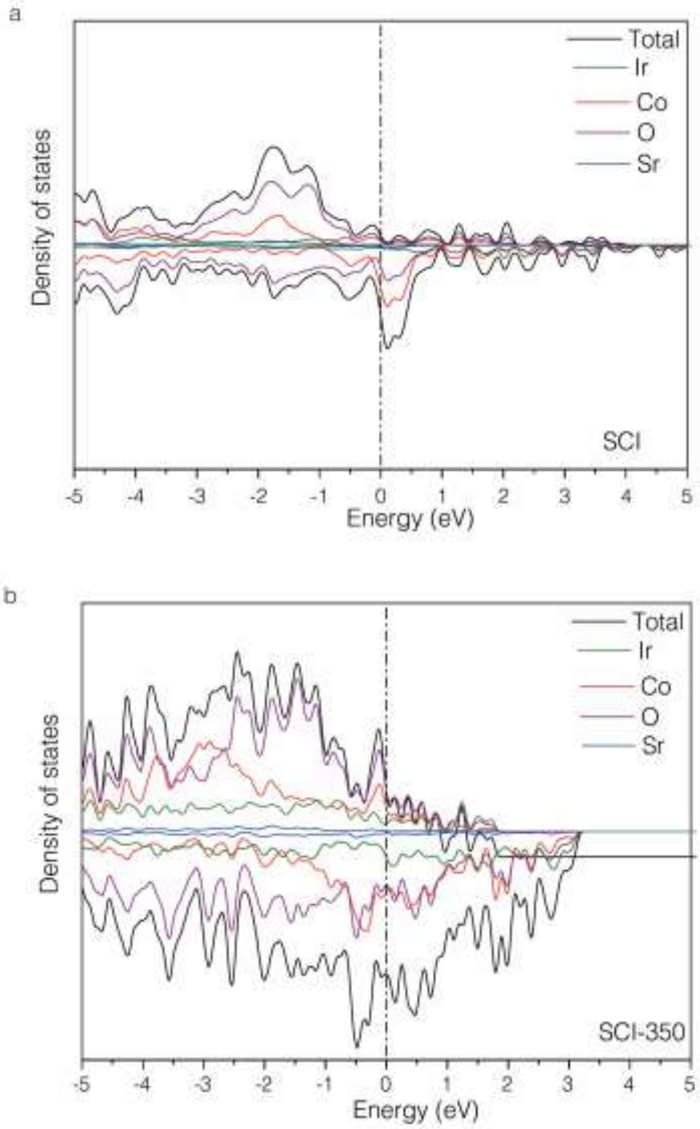

Figure S28. The PDOS of (a) SCI and (b) SCI-350

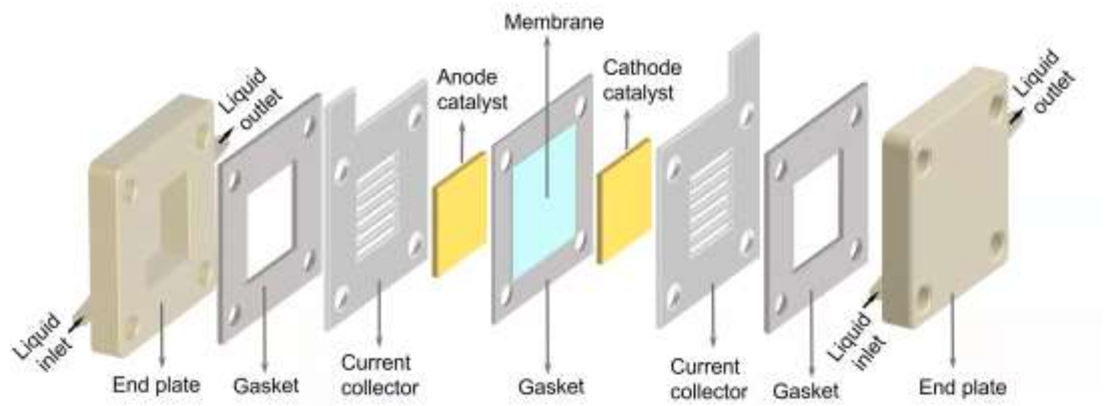

Figure S29. The schematic of water electrolyzer cell. The synthesis procedure for the preparation of a CoP/NF electrode could be found as follows. First, $Co(NO_3)_2{\bullet}6H_2O$ (0.375 mmol), $CO(NH_2)_2$ (1.875 mmol) and NH_4F ((1.5 mmol) were weighed and dissolved in 15 ml DI water. Then, the mixed solution was stirred for 20 minutes. After that, we placed the pink solution and cleaned Ni foam (with a dimension of 1 x 2 cm) in a Teflon stainless steel autoclave (50 mL) and kept warm at 120 °C for 12 hours. Finally, $NaH_2PO_2{\bullet}H_2O$ and $Co(OH)_2$/NF (mass ratio of $NaH_2PO_2{\bullet}H_2O$ to $Co(OH)_2$ was 5:1) were placed in the upper and lower reaches of quartz tube under argon atmosphere, respectively. The quartz tube was heated to 300 °C, which leads to thermal decomposition of NaH_2PO_2 and the formation of PH_3 gas. The quartz tube was kept at 300 °C for 120 minutes before being cooled to room temperature.

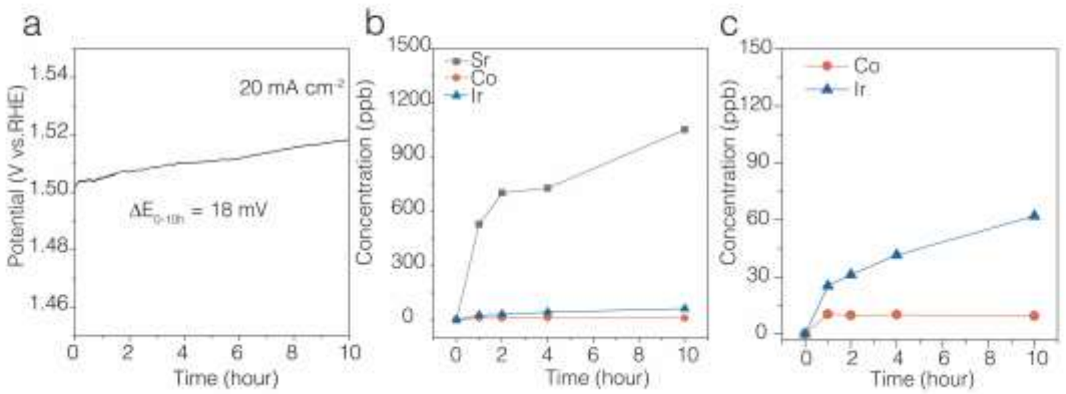

Figure S30. (a) Stability test of the SCI-350 electrocatalyst at 20 mA cm^{-2} in a three-electrode setup. (b-c) Leached metal contents in the electrolyte for SCI-350 during stability measurements.

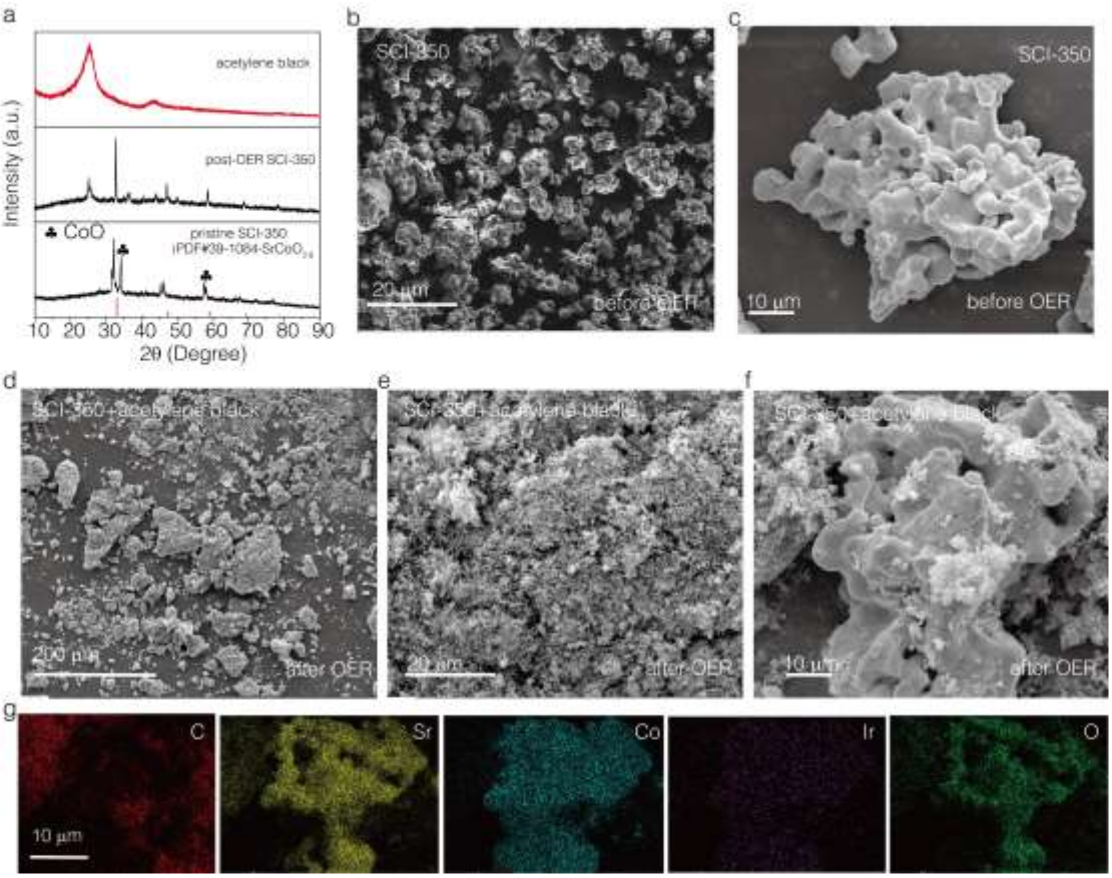

Figure S31. (a) XRD patterns of pristine SCI-350, post-OER SCI-350 and acetylene black. SEM image of (b-c) pristine SCI-350 and (d-f) post-OER SCI-350. (g) The corresponding element mappings of C, Sr, Co, Ir, and O in post-OER SCI-350.

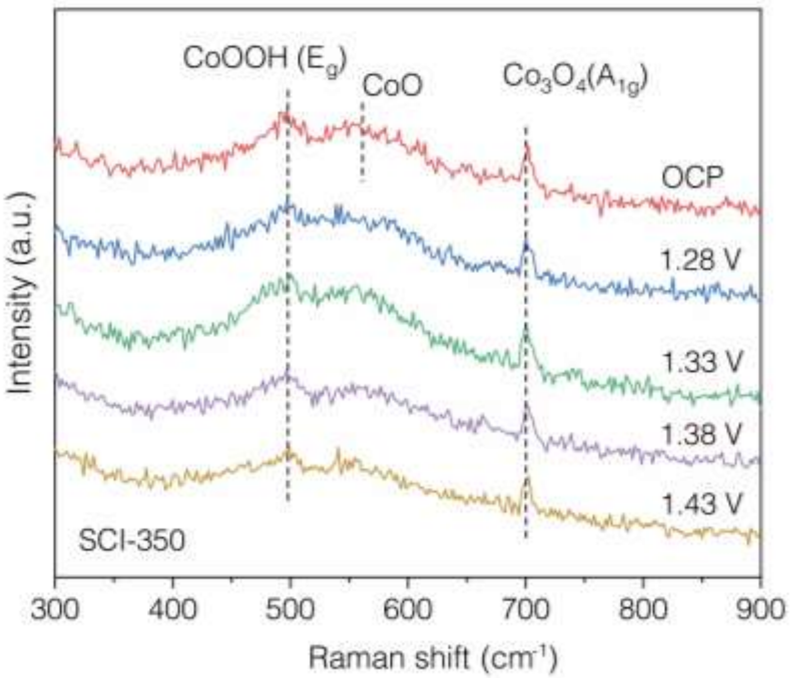

Figure S32. *In-situ* Raman spectra of SCI-350 in 1M KOH. The operando technique was employed to identify the evolution of active site of the electrocatalyst during OER. The emergence of CoO, CoOOH (E_g) and Co_3O_4 (A_{1g}) peaks were observed at the OCP condition.[5] As the potential was increased to 1.43 V, intensities of the CoOOH (E_g) and Co_3O_4 (A_{1g}) peaks decrease, probably due to the formation of large amount of oxygen molecule during OER operation.

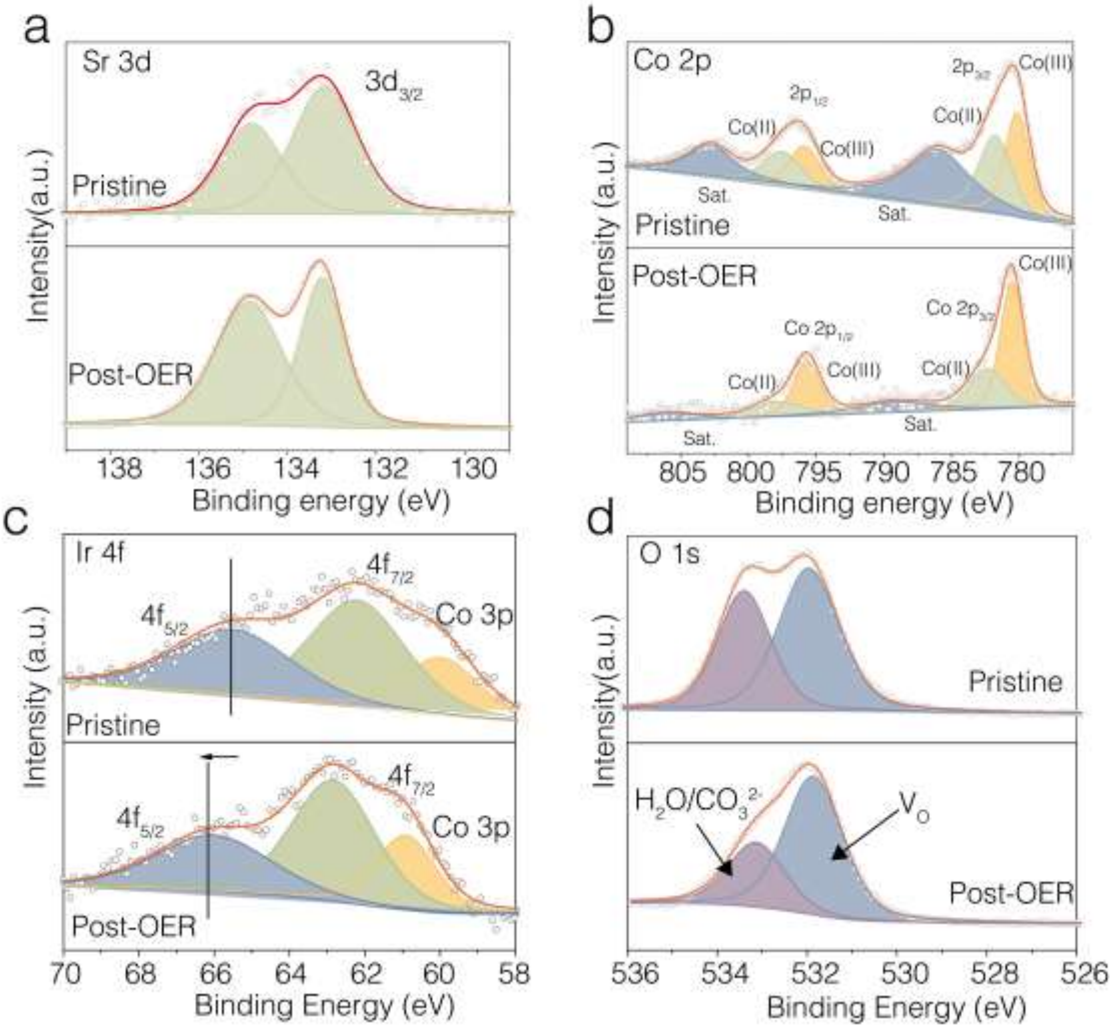

Figure S33. XPS spectra of Sr 3d, Co 2p, Ir 4f and O 1s of the pristine SCI-350 comparing with the SCI-350 after 10 h OER test at 20 mA cm^{-2} (Post-OER).

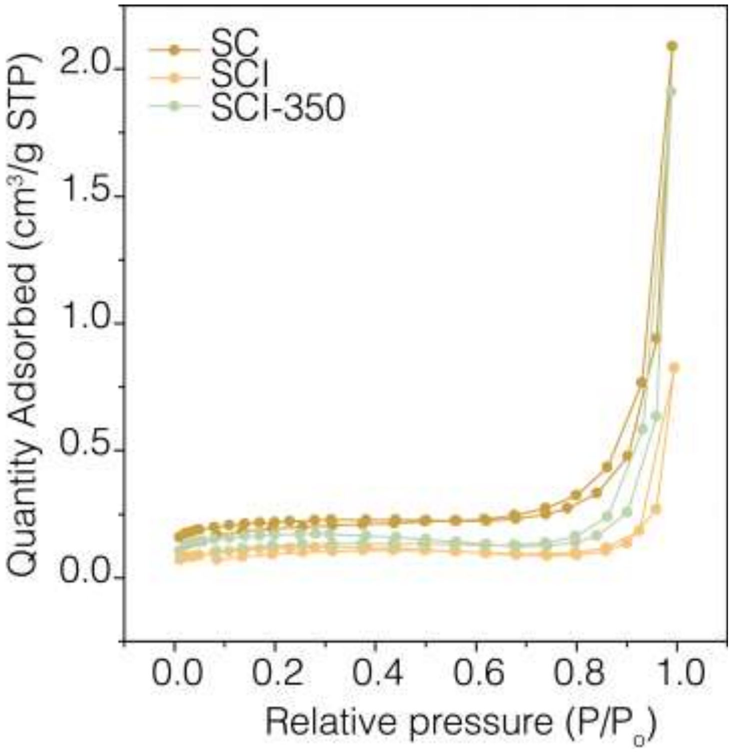

Figure S34. N_2 adsorption-desorption isotherms of SC, SCI, and SCI-350.

Table S1. L_{III}-edge EXAFS fitting results of Ir foil, IrO_2, SCI and SCI-350

Sample	Path	CN	R (Å)	σ^2 (Å^2)	R-factor
SCI	Ir-O	5.8(5)	1.94(1)	0.0025(7)	0.0391
	Ir-Sr	8.3(1)	3.37(1)	0.0070(14)	
	Ir-Co	3.3(7)	3.65(2)	0.0025(27)	
	Ir-Co	2.6(9)	4.05(3)	0.0037(29)	
SCI-350	Ir-O	3.6(1)	1.97(1)	0.0042(21)	0.0124
	Ir-Ir	6.9(4)	2.65(3)	0.0117(47)	
	Ir-Sr	1.4(3)	3.35(3)	0.0124(15)	
	Ir-Co	6.8(7)	3.68(3)	0.0091(10)	
Ir	Ir-Ir	**12**	2.70(1)	0.0059(5)	0.0337
	Ir-Ir	**6**	3.82(2)	0.0054(21)	
IrO_2	Ir-O	**6**	1.97(1)	0.0037(2)	0.0076
	Ir-Ir	**2**	3.12(1)	0.0025(7)	
	Ir-O	**8**	3.60(2)	0.0100[a]	
	Ir-Ir	**8**	3.54(1)	0.0060(5)	

The amplitude reduction factor S_0^2 and *k*-range were set to 0.95 and 3-12 Å^{-1}.

[a]Parameters held constant during fitting. Bold values were fixed in the fits.

Table S2-1. The comparison of OER performance of representative electrocatalysts.

Catalyst	Overpotential (mV) at 10 mA cm^{-2}	Ir mass Activity (A g^{-1})	Electrolyte	Tafel (mV Dec^{-1})	Mass loading (mg cm^{-2})	Substrates	Publication year	ref
SCI-350	240	597.6 at 1.5 V	1 M KOH	44	0.51	RDE		This work
La_2LiIrO_6	270	~1 at 1.73 V	0.1 M $HClO_4$	50		RDE	2016	6
$SrCo_{0.9}Ir_{0.1}O_3$	270		0.1 M $HClO_4$		0.25	RDE	2019	4
$Ba_{0.9}Sr_{0.1}Co_{0.8}Fe_{0.1}Ir_{0.1}O_{3-\delta}$	300	309.6 at 1.53 V	1 M KOH	61	0.43	RDE	2020	7
Ir nanoparticles coupled with FeP/FeOOH	169	8.65 at 1.53 V	1 M KOH	51		Fe foil	2021	8
Ir NWs	250		1 M KOH	74		RDE	2020	9
Ir@Co NSs	273		1 M KOH	99	0.30	GC	2019	10
IrO_2/CNT	249		1 M KOH	32		GC	2017	11
NiVIr LDH	203		1 M KOH	55	1.50	Ni foam	2019	12
FeIr/NF	200 at 20 mA cm^{-2}		1 M KOH	49.		Ni foam	2020	13
$Ni_{0.93}Ir_{0.07}$/rGO	271.8	473.7 at 1.504 V	1 M KOH	56		CFP	2020	14
$Ir_{0.71}Cu_{0.29}$	335	~112 at 1.565 V	1 M NaOH		0.51	RDE	2018	15
IrO_x	320		1 M NaOH			RDE	2013	16
Li-$IrSe_2$	270		1 M KOH		1.00	CFP	2019	17
$SrIrO_3$ NFs	390		1 M KOH	31	0.56	RDE	2022	18
IrO_2	330		0.1 M KOH	69	0.51	RDE	2022	19
Ir@Co_3O_4	280		0.1 M KOH	73	0.51	RDE	2022	19
$SrIrO_3$ monoclinic	300	90 at 1.55 V	0.1 M KOH	42	0.23	RDE	2020	20
α-Li_2IrO_3	290	26.45 at 1.52 V	0.1 M KOH	50	0.51	RDE	2020	21
SrIrO3(100)P/DyScO3(110)	400		0.1 M KOH	40		Thin film	2016	22
Ir@Co_4N NFs	310	106.8 at 1.51 V	0.1 M KOH	24	0.29	GC	2018	23

Table S2-2. The performance of the catalysts in this work.

Catalyst	η_{10} (mV)	Tafel (mV Dec^{-1})	C_{dl} (mF cm^{-2})	ECSA (cm^2)
SC	450	85	0.06	
$Sr_{0.9}CoO_{3-\delta}$	450	77	1.1	
$Sr_{0.9}CoO_{3-\delta}$-350	390	71	20.2	
$SrCo_{0.99}Ir_{0.01}O_{3-\delta}$	418	79		
$SrCo_{0.95}Ir_{0.05}O_{3-\delta}$	397	77		
$SrCo_{0.90}Ir_{0.1}O_{3-\delta}$	370	72		
$SrCo_{0.85}Ir_{0.15}O_{3-\delta}$	413	84		
$SrCo_{0.90}Ir_{0.1}O_{3-\delta}$-300	270	64		
$Sr_{0.95}Co_{0.90}Ir_{0.1}O_{3-\delta}$-300	269	63		
$Sr_{0.9}Co_{0.90}Ir_{0.1}O_{3-\delta}$-300	255	52	21.9	547.5
$Sr_{0.85}Co_{0.90}Ir_{0.1}O_{3-\delta}$-300	268	57		
$Sr_{0.80}Co_{0.90}Ir_{0.1}O_{3-\delta}$-300	265	62		
$SrCo_{0.90}Ir_{0.1}O_{3-\delta}$	394	82	1.5	37.5
$SrCo_{0.90}Ir_{0.1}O_{3-\delta}$-250	380	82	2.7	67.5
$SrCo_{0.90}Ir_{0.1}O_{3-\delta}$-350	240	44	49.2	1230
$SrCo_{0.90}Ir_{0.1}O_{3-\delta}$-400	250	51	28.6	715

Table S3-1. The content of each element measured by different tools.

SCI	SEM-EDX	XPS	ICP	*Raw materials
Sr	37.19 wt%	38.12 wt%	39.22 wt%	39.60 wt%
Co	25.11 wt%	25.38 wt%	24.64 wt%	26.36 wt%
Ir	10.80 wt%	9.45 wt%	9.82 wt%	9.65 wt %

SCI-350	SEM-EDX	XPS	ICP	*Raw materials
Sr	39.76 wt%	39.78 wt%	40.88 wt%	39.60 wt%
Co	27.24 wt%	26.42 wt%	28.82 wt%	26.36 wt%
Ir	9.02 wt%	10.12 wt%	9.46 wt%	9.65 wt%

*The Raw materials means the weight percentage of the precursor powders during preparation.

Table S3-2. The content of each element measured by different tools.

Sample	wt% of Ir
IrO_2	85.7%
$SrIrO_3$[4]	58.6%
La_2LiIrO_6[6]	33.5%
$SrCo_{0.9}Ir_{0.1}O_3$	9.3%

Table S3-3. The relative ratio of Co(II) to Co(III) in SCI and SCI-350 from XPS spectra.

Sample	Co^{2+} (%)	Co^{3+} (%)	Average valence
SCI	39.4	60.6	2.61
SCI-350	49.6	50.4	2.50

Table S4. The BET surface area of various samples.

Sample	BET surface area (m^2 g^{-1})
SC	0.694
SCI	0.378
SCI-350	0.536

Table S5. Summary of the impedance fitting data for SCI electrode in 1M KOH.

Potential (V vs.RHE	R_s (Ω)	T_1 (S $s^{\alpha1}$)	R1 (Ω)	T_2 (S $s^{\alpha2}$)	R_{ct} (Ω)	α1	α2
1.45	5.663	0.005666	1.394	0.001021	2005	0.59	0.95
1.50	5.636	0.01214	1.123	0.000974	263.1	0.54	0.89
1.55	5.659	6.921E-5	1.102	0.00114	98.63	0.78	1
1.60	5.664	0.0009011	1.54	0.00274	26.96	0.66	1
1.65	5.715	0.07279	1.33	0.001021	12.53	0.62	1
1.70	5.781	0.2395	1.152	0.0011	6.092	0.76	0.74

Table S6. Summary of the impedance fitting data for SCI-350 electrode in 1 M KOH.

Potential (V vs.RHE)	R_s (Ω)	T_1 (S $s^{\alpha1}$)	R_1 (Ω)	T_2 (S $s^{\alpha2}$)	R_{ct} (Ω)	α1	α2
1.45	6.046	0.0009841	1.996	0.009167	99.55	0.8	0.9
1.50	5.877	0.003858	2.141	0.007986	18.37	0.8	0.8
1.55	6.079	0.0001114	1.267	0.009579	10.1	0.8	0.73
1.60	6.124	2.95E-5	1.048	0.01139	7.077	0.8	0.8
1.65	6.288	1.843E-5	0.9123	0.01193	5.264	1	0.63
1.70	6.307	1.453E-8	0.9586	0.01569	4.217	0.95	0.57

Table S7. The oxygen diffusion coefficient of various samples.

Sample	D_O (cm² s^{-1})
SC	2.96×10^{-11}
SCI	3.08×10^{-11}
SCI-350	10.58×10^{-11}

Table S8. The comparison of AWE performance of state-of-art electrocatalysts.

Type	Catalyst	Electrolyte	Temperature/^{o}C	Performance	Stability	Reference
Zero-gap	SCI-350(+) || Cobalt phosphide(-)	6 M KOH	25	1.95 V	0.5 A cm^{-2}	This work
				1A cm^{-2}	150 h	
AWE	$CoFe_xAl_{2-x}O_4$(+) || Pt/C(-)	0.1 M KOH	60	1.96 V		[24]
				1 A cm^{-2}		
AWE	NiFeCo LDH(+) || NiFeCo phosphide(-)	1 M KOH	50	1.75 V	0.5 A cm^{-2}	[25]
				0.5 A cm^{-2}	40 h	
AWE	NiFeMo(+) || Ru(-)	30% KOH	85	~1.7 V	0.3 A cm^{-2}	[26]
				0.3 A cm^{-2}	120 h	
AWE	Ni/Mo-Ni(+) || Ni/Mo-Ni(-)	1 M KOH	25	1.76 V	0.1 A cm^{-2}	[27]
				0.1 A cm^{-2}	87h	
AWE	Mo-NiPx/NiSy(+) || MoNiPx/NiSy(-)	1 M KOH	25	1.9 V	0.05 A cm^{-2}	[28]
				0.4 A cm^{-2}	20 h	
AWE	B-NiFe-LDH(+) || Pt/C(-)	1 M KOH	50	1.74 V	1 A cm^{-2}	[29]
				1A cm^{-2}	150 h	
AWE	$Cu_{0.81}Co_{2.19}O_4$(+) || Co_3S_4 NS(-)	1 M KOH	45-48	2.19 V	0.5 A cm^{-2}	[30]
				1A cm^{-2}	10 h	
AWE	$CuCoO_4$(+) || Pt/C(-)	1 M KOH	45	1.76 V	0.5 A cm^{-2}	[31]
				1A cm^{-2}	64 h	
AWE	IrO_2(+) | |Pt/C(-)	1 M KOH	45	1.8 V	0.5 A cm^{-2}	[31]
				0.76 A cm^{-2}	64 h	

Table S9. The content of each element of SCI-350 and post-OER SCI-350 measured by XPS.

	Sr	Co	Ir	O
SCI-350	39.78 wt%	26.42 wt%	10.12 wt%	23.68 wt%
Post-OER SCI-350	37.99 wt%	27.02 wt%	10.45 wt%	24.54 wt%

References

(1) Yang, L.; Yu, G.; Ai, X.; Yan, W.; Duan, H.; Chen, W.; Li, X.; Wang, T.; Zhang, C.; Huang, X. Efficient Oxygen Evolution Electrocatalysis in Acid by a Perovskite with Face-Sharing IrO6 Octahedral Dimers. *Nat. Commun.* **2018,** *9*, 1-9.

(2) Seitz, L. C.; Dickens, C. F.; Nishio, K.; Hikita, Y.; Montoya, J.; Doyle, A.; Kirk, C.; Vojvodic, A.; Hwang, H. Y.; Norskov, J. K. A highly Active and Stable IrO x/SrIrO3 Catalyst for the Oxygen Evolution Reaction. *Science* **2016,** *353*, 1011-1014.

(3) Li, J.; Lu, G.; Wu, G.; Mao, D.; Guo, Y.; Wang, Y.; Guo, Y. The Role of Iron Oxide in the Highly Effective Fe-Modified Co3O4 Catalyst for Low-Temperature CO Oxidation. *RSC Adv.* **2013,** *3*, 12409-12416.

(4) Chen, Y.; Li, H.; Wang, J.; Du, Y.; Xi, S.; Sun, Y.; Sherburne, M.; Ager, J. W., 3rd; Fisher, A. C.; Xu, Z. J. Exceptionally Active Iridium Evolved from a Pseudo-Cubic Perovskite for Oxygen Evolution in Acid. *Nat. Commun.* **2019,** *10*, 572.

(5) Lee, W. H.; Han, M. H.; Ko, Y.-J.; Min, B. K.; Chae, K. H.; Oh, H.-S. Electrode Reconstruction Strategy for Oxygen Evolution Reaction: Maintaining Fe-CoOOH Phase with Intermediate-Spin State during Electrolysis. *Nat. Commun.* **2022,** *13*, 605.

(6) Grimaud, A.; Demortière, A.; Saubanère, M.; Dachraoui, W.; Duchamp, M.; Doublet, M.-L.; Tarascon, J.-M. Activation of Surface Oxygen Sites on an Iridium-Based Model Catalyst for the Oxygen Evolution Reaction. *Nat. Energy* **2016,** *2,* 16189.

(7) Luo, Q.; Lin, D.; Zhan, W.; Zhang, W.; Tang, L.; Luo, J.; Gao, Z.; Jiang, P.; Wang, M.; Hao, L.; Tang, K. Hexagonal Perovskite Ba0.9Sr0.1Co0.8Fe0.1Ir0.1O3−δ as an Efficient Electrocatalyst towards the Oxygen Evolution Reaction. *ACS Appl. Energy Mater.* **2020,** *3*, 7149-7158.

(8) Zhao, Z.; Jin, W.; Xu, L.; Wang, C.; Zhang, Y.; Wu, Z. Ultrafine Ir Nanoparticles Decorated on FeP/FeOOH with Abundant Interfaces via a Facile Corrosive Approach for Alkaline Water-Splitting. *J. Mater. Chem. A* **2021,** *9*, 12074-12079.

(9) Chen, Z.; Duan, X.; Wei, W.; Wang, S.; Ni, B.-J. Iridium-Based Nanomaterials for Electrochemical Water Splitting. *Nano Energy* **2020,** *78,* 105392.

(10) Babu, D. D.; Huang, Y.; Anandhababu, G.; Wang, X.; Si, R.; Wu, M.; Li, Q.; Wang, Y.; Yao, J. Atomic Iridium@Cobalt Nanosheets for Dinuclear Tandem Water

Oxidation. *J. Mater. Chem. A* **2019,** *7*, 8376-8383.

(11) Guan, J.; Li, D.; Si, R.; Miao, S.; Zhang, F.; Li, C. Synthesis and Demonstration of Subnanometric Iridium Oxide as Highly Efficient and Robust Water Oxidation Catalyst. *ACS Catal.* **2017,** *7*, 5983-5986.

(12) Li, S.; Xi, C.; Jin, Y.-Z.; Wu, D.; Wang, J.-Q.; Liu, T.; Wang, H.-B.; Dong, C.-K.; Liu, H.; Kulinich, S. A.; Du, X.-W. Ir–O–V Catalytic Group in Ir-Doped NiV(OH)2 for Overall Water Splitting. *ACS Energy Lett.* **2019,** *4*, 1823-1829.

(13) Shen, F.; Wang, Y.; Qian, G.; Chen, W.; Jiang, W.; Luo, L.; Yin, S., Bimetallic Iron-Iridium Alloy Nanoparticles Supported on Nickel Foam as Highly Efficient and Stable Catalyst for Overall Water Splitting at Large Current Density. *Appl. Catal. B Environ.* **2020,** *278,* 119327.

(14) Zhang, S.; Zhang, X.; Shi, X.; Zhou, F.; Wang, R.; Li, X. Facile Fabrication of Ultrafine Nickel-iridium Alloy Nanoparticles/Graphene Hybrid with Enhanced Mass Activity and Stability for Overall Water Splitting. *J. Energy Chem.* **2020,** *49*, 166-173.

(15) Wang, F.; Kusada, K.; Wu, D.; Yamamoto, T.; Toriyama, T.; Matsumura, S.; Nanba, Y.; Koyama, M.; Kitagawa, H., Solid-Solution Alloy Nanoparticles of the Immiscible Iridium-Copper System with a Wide Composition Range for Enhanced Electrocatalytic Applications. *Angew. Chem. Int. Ed. Engl.* **2018,** *57*, 4505-4509.

(16) McCrory, C. C.; Jung, S.; Peters, J. C.; Jaramillo, T. F. Benchmarking Heterogeneous Electrocatalysts for the Oxygen Evolution Reaction. *J. Am. Chem. Soc.* **2013,** *135*, 16977-87.

(17) Zheng, T.; Shang, C.; He, Z.; Wang, X.; Cao, C.; Li, H.; Si, R.; Pan, B.; Zhou, S.; Zeng, J. Intercalated Iridium Diselenide Electrocatalysts for Efficient pH-Universal Water Splitting. *Angew. Chem. Int. Ed. Engl.* **2019,** *58*, 14764-14769.

(18) Shin, S.; Kwon, T.; Kim, K.; Kim, M.; Kim, M. H.; Lee, Y. Single-Phase Perovskite SrIrO3 Nanofibers as a Highly Efficient Electrocatalyst for a pH-Universal Oxygen Evolution Reaction. *ACS Appl. Energy Mater.* **2022,** *5*, 6146-6154.

(19) Dai, Y.; Yu, J.; Wang, J.; Shao, Z.; Guan, D.; Huang, Y. C.; Ni, M. Bridging the Charge Accumulation and High Reaction Order for High-Rate Oxygen Evolution and

Long Stable Zn-Air Batteries. *Adv. Func. Mater.* **2022,** 32, 2111989.

(20) Yu, J.; Wu, X.; Guan, D.; Hu, Z.; Weng, S.-C.; Sun, H.; Song, Y.; Ran, R.; Zhou, W.; Ni, M.; Shao, Z. Monoclinic SrIrO3: An Easily Synthesized Conductive Perovskite Oxide with Outstanding Performance for Overall Water Splitting in Alkaline Solution. *Chem. Mater.* **2020,** *32*, 4509-4517.

(21) Yang, C.; Rousse, G.; Louise Svane, K.; Pearce, P. E.; Abakumov, A. M.; Deschamps, M.; Cibin, G.; Chadwick, A. V.; Dalla Corte, D. A.; Anton Hansen, H.; Vegge, T.; Tarascon, J. M.; Grimaud, A. Cation Insertion to Break the Activity/Stability Relationship for Highly Active Oxygen Evolution Reaction Catalyst. *Nat. Commun.* **2020,** *11*, 1378.

(22) Tang, R.; Nie, Y.; Kawasaki, J. K.; Kuo, D.-Y.; Petretto, G.; Hautier, G.; Rignanese, G.-M.; Shen, K. M.; Schlom, D. G.; Suntivich, J. Oxygen Evolution Reaction Electrocatalysis on SrIrO3 Grown Using Molecular Beam Epitaxy. *J. Mater. Chem. A* **2016,** *4*, 6831-6836.

(23) Cho, S.-H.; Yoon, K. R.; Shin, K.; Jung, J.-W.; Kim, C.; Cheong, J. Y.; Youn, D.-Y.; Song, S. W. Henkelman, G.; Kim, I.-D. Synergistic Coupling of Metallic Cobalt Nitride Nanofibers and IrOx Nanoparticle Catalysts for Stable Oxygen Evolution. *Chem. Mater.* **2018,** *30*, 5941-5950.

(24) Wu, T.; Sun, S.; Song, J.; Xi, S.; Du, Y.; Chen, B.; Sasangka, W. A.; Liao, H.; Gan, C. L.; Scherer, G. G.; Zeng, L.; Wang, H.; Li, H.; Grimaud, A.; Xu, Z. J. Iron-Facilitated Dynamic Active-site Generation on Spinel CoAl2O4 with Self-Termination of Surface Reconstruction for Water Oxidation. *Nat. Catal.* **2019,** *2*, 763-772.

(25) Lee, J.; Jung, H.; Park, Y. S.; Kwon, N.; Woo, S.; Selvam, N. C. S.; Han, G. S.; Jung, H. S.; Yoo, P. J.; Choi, S. M.; Han, J. W.; Lim, B. Chemical Transformation Approach for High-Performance Ternary NiFeCo Metal Compound-Based Water Splitting Electrodes. *Appl. Catal. B Environ.* **2021,** *294,* 120246.

(26) Zhang, B.; Wang, L.; Cao, Z.; Kozlov, S. M.; García de Arquer, F. P.; Dinh, C. T.; Li, J.; Wang, Z.; Zheng, X.; Zhang, L.; Wen, Y.; Voznyy, O.; Comin, R.; De Luna, P.; Regier, T.; Bi, W.; Alp, E. E.; Pao, C.-W.; Zheng, L.; Hu, Y.; Ji, Y.; Li, Y.; Zhang, Y.;

Cavallo, L.; Peng, H.; Sargent, E. H. High-Valence Metals Improve Oxygen Evolution Reaction Performance by Modulating 3d Metal Oxidation Cycle Energetics. *Nat. Catal.* **2020,** *3*, 985-992.

(27) Li, H.; Cai, C.; Wang, Q.; Chen, S.; Fu, J.; Liu, B.; Hu, Q.; Hu, K.; Li, H.; Hu, J.; Liu, Q.; Chen, S.; Liu, M. High-performance Alkaline Water Splitting by Ni Nanoparticle-Decorated Mo-Ni Microrods: Enhanced Ion Adsorption by the Local Eectric Field. *Chem. Eng. J.* **2022,** *435,* 134860.

(28) Wang, J.; Zhang, M.; Yang, G.; Song, W.; Zhong, W.; Wang, X.; Wang, M.; Sun, T.;Tang, Y. Heterogeneous Bimetallic Mo-NiPx/NiSy as a Highly Efficient Electrocatalyst for Robust Overall Water Splitting. *Adv. Funct. Mater.* **2021,** *31,* 2101532.

(29) Jeon, S. S.; Lim, J.; Kang, P. W.; Lee, J. W.; Kang, G.; Lee, H., Design Principles of NiFe-Layered Double Hydroxide Anode Catalysts for Anion Exchange Membrane Water Electrolyzers. *ACS Appl Mater Interfaces* **2021,** *13*, 37179-37186.

(30) Park, Y. S.; Lee, J. H.; Jang, M. J.; Jeong, J.; Park, S. M.; Choi, W.-S.; Kim, Y.; Yang, J.; Choi, S. M. Co3S4 Nanosheets on Ni Foam via Electrodeposition with Sulfurization as Highly Active Electrocatalysts for Anion Exchange Membrane Electrolyzer. *Int. J. Hydrogen Energy* **2020,** *45*, 36-45.

(31) Park, Y. S.; Yang, J.; Lee, J.; Jang, M. J.; Jeong, J.; Choi, W.-S.; Kim, Y.; Yin, Y.; Seo, M. H.; Chen, Z.; Choi, S. M. Superior Performance of Anion Exchange Membrane Water Electrolyzer: Ensemble of Producing Oxygen Vacancies and Controlling Mass Transfer Resistance. *Appl. Catal. B Environ.* **2020,** *278,* 119276.
